# Evolutionary and ecological success is decoupled in mammals

**DOI:** 10.1111/jbi.13411

**Published:** 2018-07-31

**Authors:** Søren Faurby, Alexandre Antonelli

**Affiliations:** ^1^ Department of Biological and Environmental Sciences University of Gothenburg Göteborg Sweden; ^2^ Gothenburg Global Biodiversity Centre Göteborg Sweden; ^3^ Gothenburg Botanical Garden Göteborg Sweden; ^4^ Department of Organismic and Evolutionary Biology Harvard University Cambridge Massachusetts

**Keywords:** adaptability, colonization, dispersal limitation, ecological success, mammals, range size

## Abstract

**Aim:**

To identify which factors distinguish ecologically successful mammalian clades (i.e., clades with a large combined range size) from less successful ones.

**Location:**

Global.

**Methods:**

We estimated the total range sizes for each individual mammalian subfamily and used phylogenetic regressions to identify the relative importance of factors related to colonization ability (body size and niche width) and adaptability (rate of evolution of body size and rate of evolution of climatic preference) in determining these ranges. We then estimated the importance of the same factors on the variation in diversification rate within mammals.

**Results:**

We found strong support for a link between total range size and traits related to colonization ability. In particular, we found larger total range sizes among clades containing larger bodied species and clades with wider climatic niche width, while we did not find support for any predictors related to adaptability being linked to total range size. We also found that traits related to increased range size were associated with reduced diversification rate.

**Main Conclusions:**

Range size for mammalian clades is mainly predicted by colonization ability, suggesting that most clades are limited by dispersal rather than their ability to adapt to new environments. The most ecologically successful (i.e., most widespread) mammalian clades tend to possess traits that reduce geographical isolation among populations, but the same traits tend to decrease diversification rates. Our results unveil a decoupling between evolutionary and ecological success in mammals.

## 1 INTRODUCTION

The word “success” abounds in human communication, but it has also been widely used in biodiversity research. Only in very few cases, however, is it easy to define what clade is most “successful”—almost irrespective of how one would define ecological or evolutionary success. For instance, few would argue that the tetrapods are not more successful than their sister group, the Sarcopterygii (lungfishes and coelacanths). In most cases, however, it is less clear which clades are the most successful ones. To address this question, many studies have taken a macroevolutionary perspective by assessing which clades show the highest species diversity or the highest diversification rates and therefore have highest “evolutionary success” (e.g., Alfaro et al., 2009). “Ecological success”, in contrast, may be attributed to clades with higher ecological importance—in terms of number of individuals, biomass or spatial and/or niche occupancy. For instance, krill (Euphausiacea) can be considered extremely successful since they are some of the most abundant of all animals (Atkinson, Siegel, Pakhomov, Jessopp, & Loeb, 2009)—even though they are believed to comprise less than one hundred species (Spiridonov & Casanova, 2010). Another straightforward way of defining ecologically successful clades, and the one considered here, is to consider the size of their geographical range.

An important caveat when discussing clade success and in particular the traits associated with it is that some traits (like body size) are only defined at the individual level, whereas others (like rates of change) are arguably only defined at the species level. The discussion of traits characterizing even higher phylogenetic levels, involving entire species clades, is therefore even more challenging. However, this is justifiable if higher‐level clades are real biological entities as has been suggested from an evolutionary viewpoint (e.g., Humphreys & Barraclough, 2014), and as long as traits have a strong phylogenetic signal. For simplicity, here we discuss traits in the context of clades, even though we are more specifically referring to the means of the species within clades.

Different definitions of clade success require the consideration of different underlying factors. In some cases, different definitions of clade success may be in direct opposition to each other. An example is the lagomorph genus *Prolagus* (Fostowicz‐Frelik, 2010), which survived in isolated relictual parts of its formerly widespread range and finally went extinct from its last populations in Corsica and Sardinia due to humans (IUCN, 2015). The genus reached its highest diversity in the Pliocene, but the largest geographical range in the Miocene; the higher diversity in the Pliocene was probably just a consequence of increased allopatric speciation due to lower gene flow (Fostowicz‐Frelik, 2010). In such cases, ecological and evolutionary success may constitute alternative views of when taxa were most successful.

There is a potential but unexplored connection between ecological and evolutionary success. For instance, consider two hypothetical clades that only differ in their number of species. If the species’ climatic preferences change in accordance with a Brownian motion model (Felsenstein, 1973), the clade with more species will most likely have the largest total range. Conversely, a larger total range size for otherwise identical clades should provide more opportunity for speciation, as larger areas usually have more biological barriers (such as a rivers and mountains, for terrestrial organisms) that can increase the probability of allopatric speciation.

One class of factors potentially underlying a clade's ecological success can be grouped as colonization factors, which are associated with the ability of clades to expand their ranges. One of these is body size, which is strongly correlated with average daily movement distances (Wolf, Doughty, & Malhi, 2013) and weakly correlated with starvation times (McNab, 2010). Combined, these correlations ought to make it easier for larger species to cross barriers. Given that there is a strong phylogenetic signal in body size (Blomberg, Garland, & Ives, 2003), clades of larger bodied species may therefore typically have larger total range sizes. Niche‐related factors (here referred to in terms of realized rather than fundamental niche) might also be related to the colonization ability of species and, assuming phylogenetic signal in the relevant traits, the colonization ability of larger clades. This is most obvious in terms of niche width, as clades of generalist species (i.e., species with individually large niche widths) ought to have a larger colonization capacity (and therefore larger total range) than clades mainly containing specialist species.

Another set of factors that might determine total ranges can be grouped under adaptability, which we define here strictly in terms of evolutionary (i.e., permanent) changes rather than plastic (nonheritable) responses. Adaptability factors are associated with a clade's survival under various climatic conditions. This includes survival despite climatic changes in already occupied areas, or the ability to colonize adjacent but climatically unsuitable areas. Using our criteria, both increased colonization ability and increased adaptability may thus enable clades to colonize new areas; the former by allowing individual animals to more easily move across unsuitable areas and the latter by increasing the area suitable for occupation.

Clades with larger total ranges may generally be the ones with the largest adaptive capacity. In environmental space this is similar to the idea of niche conservatism in a broad sense (Wiens & Donoghue, 2004). Niche conservatism is often discussed in terms of the latitudinal diversity gradient, but the underlying process has particular relevance in this context. Most tropical clades that colonized colder areas also persisted in tropical areas, leading to an increase in phylogenetic diversity relative to species richness with increased annual temperature, as documented for mammals (Safi et al., 2011). Clades capable of adapting to colder areas could, therefore, generally be expected to occur in a larger total area. In the same way as climatic adaptability, morphological adaptability could also increase ecological success, given that different habitats may select for different body sizes. For mammals, higher latitudes generally select for larger body sizes (Faurby & Araújo, 2017; Meiri & Dayan, 2004), islands select for intermediate body sizes (Faurby & Svenning, 2016a; Lomolino, Sax, Palombo, & Van der Geer, 2012), and closed forests and deserts may select for small sizes, at least in some clades (McNab, 2010; Perry et al., 2014). In general, clades with a more body size adaptability could therefore be expected to colonize larger areas than clades with less body size adaptability.

A third group of potential predictors of total range size can be called historical contingencies. These constitute abiotic conditions that modified the possibilities that clades had to colonize new areas—such as changes in climatic conditions, vegetation cover (e.g., transitions between forest and savannahs) and connectivity between landmasses. For most of the Cenozoic, the world was substantially warmer than today (Herbert et al., 2016). During warmer periods warm‐adapted lineages presumably dispersed between Africa and Eurasia, and between Eurasia and North America, more frequently than they did in cooler phases (Donoghue, 2008). After North and South America came into contact after a long period of isolation many lineages dispersed between these landmasses (Bacon et al., 2015; Faurby & Svenning, 2016a,b). Given that a terrestrial connection was established across tropical Mesoamerica (Bacon et al., 2016), dispersals between the two American continents may have been especially common among warm adapted lineages (Faurby & Svenning, 2016a,b; Webb, 1991). Dispersal between Eurasia and Africa would likewise be expected to be more common for warm adapted lineages, although the fossil record here for many clades may be insufficient to determine extant and directionality of dispersal. The temperate biomes may have been more isolated. Intermittent contact occurred between the Palaearctic and Nearctic through the Bering Strait, but this was generally during the coldest periods, during which only cold‐adapted lineages were likely capable of crossing the land bridge. The combined effect is that we expect warm adapted lineages to have benefited from historical contingencies and therefore have a larger total range than cold adapted ones.

For simplicity we consider here dispersal as a symmetrical process made possible whenever formerly isolated faunas came into contact. We acknowledge, however, that formerly isolated faunas often respond differently upon contact, leading to asymmetrical dispersal rates (see e.g., Bacon et al., 2015; Pires, Silvestro, & Quental, 2015) even though the asymmetry may be less evident when focussing on a higher taxonomical level (Faurby & Svenning, 2016a,b).

**Figure 1:**
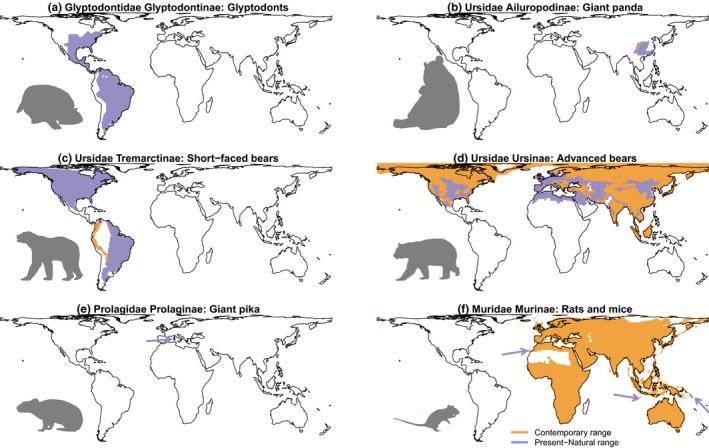
Examples of variation in the total range of clades and the extent to which these have been modified by humans. The figure shows the contemporary ranges of selected mammalian clades (following IUCN) and the present‐natural ranges (estimated ranges as inferred without any human involvement, given the contemporary climate). The ranges for the larger bodied clades (a–d) have generally been more influenced by humans than the ranges for smaller bodied clades (e–f). These modifications mean that naturally wide ranging clades, such as glyptodonts, have been driven to extinction. In some cases, the relative size of the natural range of different clades may be unidentifiable by the contemporary range, as illustrated by the short‐faced bears (which used to be widespread in the Americas but whose only surviving member is the narrowly distributed Andean spectacled bear). In contrast, the giant panda has a present‐natural range that only covers a moderate sized area of Asia, but its current distribution is similar in size to the current distribution of short‐faced bears (b–c). Some smaller bodied clades were also driven to extinction, such as the giant pika (e, natural range highlighted with an arrow), but these were narrow endemics, whereas clades with large contemporary ranges are the same as the ones with large present‐natural ones even if there were small changes (f, present‐natural range not currently occupied are highlighted with arrows) [Colour figure can be viewed at wileyonlinelibrary.com]

While these three classes of potential correlates of ecological success can be readily defined, no previous study has analysed the importance of each of them on the ecological success of clades. Here we address this problem by inferring which explanatory classes of factors have been most important in determining ecological success in mammalian subfamilies. We do this by investigating the relationship between total range size in mammal subfamilies (treating total range size as a surrogate of ecological success) and a number of traits associated with either colonization ability, adaptability or historical contingencies (Table 1). Mammals are highly suitable for pursuing this goal as there is substantial variation in the range size of different mammalian clades. Moreover, although there has been substantial human modification of the ranges of many mammalian clades, mammals are one of the only clades where we have information on the present‐natural ranges (i.e., the ranges as they would be based on contemporary climate but without any additional human involvement aside from anthropogenic global warming; Faurby & Svenning, 2015a). This means that analyses focusing on mammals, unlike most other clades, can be conducted on these potential ranges rather than on the human‐biased actual ranges—which is important given the substantial anthropogenic contractions of range sizes of many clades (Figure 1).

**Table 1:** Mechanistic explanation of the tested predictors

Proxy for	Predictor	Potential mechanism
Colonization	Body size	Higher dispersal capacity of larger animals
	Temperature niche width	Large niche width should increase the ability to cross environmental barriers
	Precipitation niche width	As above
Adaptability	Rate of body size evolution	Different body sizes are selected for under different climatic conditions. Clades with high rate of body size evolution may adapt to more variable climates and therefore occupy a larger total area
	Rate of temperature preference evolution	Clades with faster evolution of climatic preferences may have been able to adapt to a wider set of climates and therefore occupy a larger total area
	Rate of precipitation preference evolution	As above
Historical contingencies	Annual temperature	Tropical regions have historically been more inter‐connected than extra‐tropical ones. Dispersal across regions may have been easier for warm‐adapted lineages
	Annual precipitation	The connection between several of the world's biogeographical realms is through dry conditions (e.g., the Sahara) and dispersal may therefore be easier for dry‐adapted lineages
Nuisance parameter	Number of species	If species are randomly distributed, clades with more species will on average have a larger total range

## 2 MATERIALS AND METHODS

### 2.1 Input data

Analyses were restricted to nonmarine taxa and conducted at the subfamily level with some subfamilies modified to ensure monophyly. Our starting point was 244 subfamilies, but two were excluded due to lack of body size data (see Additional methods: Taxonomy and Tables S3‐4). We analysed total geographical ranges of subfamilies on an equal area Behrman projection with a column width of 1° (and therefore an area of ~9,300 km^2^) and calculated the number of cells containing at least one species. We used the geographical range data of Faurby and Svenning (2015a; using V 1.1000 available from http://bios.au.dk/en/about-bioscience/organisation/ecoinformatics-and-biodiversity/data/). These were based on the IUCN geographical ranges (IUCN, 2015), but incorporate present‐natural geographical ranges for any species with a strong indication of anthropogenic range modification, as well as any species that went extinct during the Late Pleistocene or Holocene. We thereby assumed an anthropogenic causation of all such extinctions (Sandom, Faurby, Sandel, & Svenning, 2014; Bartlett et al., 2016; but see Cooper et al., 2015), as it is notoriously difficult to distinguish the supposedly few natural Late Pleistocene extinctions from the substantially more numerous anthropogenic ones.

### 2.2 Potential predictors

We tested the correlations between traits likely associated with (a) colonization (body size, temperature niche width and precipitation niche width), (b) adaptability (rate of body size evolution, rate of temperature preference evolution and rate of precipitation preference evolution), and (c) historical contingencies (annual temperature and annual precipitation) in a multiple regression framework (see Table 1). We also included the number of species in the models but treated it as a confounding factor rather than a parameter of specific interest, because we could not identify the direction of any recovered correlation between range size and species diversity.

Details on how each predictor was calculated can be found in the Additional Methods (under Parameter estimation), but in short we estimated niche width based on quantiles rather than on entire ranges. This means that range size and niche width are not automatically correlated, which would be the case had we used the full range of values, as maximum values will depend on sample size for any distribution (see also Additional Methods: Quantile justification). Range size and six of the predictor variables (Number of species, Temperature niche width, Precipitation niche width, Rate of body size evolution, Rate of temperature preference evolution and Rate of precipitation preference evolution) were log‐transformed to improve normality (which was assessed through manual inspection of the histograms). All 10 predictors were standardized to have a mean of 0 and a standard deviation of 1.

For the analyses we assumed that the traits can meaningfully be assigned to the entire subfamily. To test this assumption we estimated the phylogenetic signal of the traits based on Pagels's lambda (Pagel, 1999; measured at the subfamily level) for 1,000 trees from Faurby and Svenning (2015b) using v. 1.1000, of their phylogeny (available from http://bios.au.dk/en/about-bioscience/organisation/ecoinformatics-and-biodiversity/data/).

In order to further assess drivers of total range size, we investigated spatial and taxonomic patterns in the residuals from the models (for residual values by subfamily see Table S5). We mainly used these residuals to investigate effects of historical contingencies by evaluating whether lineages in different continents consistently have smaller or larger ranges than estimated based on the models. We also identified clades having larger or smaller ranges than predicted by the models, in order to infer plausible underlying factors.

### 2.3 Regression analyses

Total geographical ranges of each subfamily as a function of the nine potential predictors were analysed using phylogenetic generalized least squares (PGLS) regression (Freckleton, Harvey, & Pagel, 2002). We analysed each tree in the sample of 1,000 trees from the posterior distribution from Faurby and Svenning (2015b) using v. 1.1000 giving equal weight to the results from each tree. We tested multiple models of evolution of range size in the PGLS analyses (Brownian motion, Ornstein‐Uhlenbeck (OU), Early Burst and Pagel's lambda, delta and kappa). Based on median AIC values for the 1,000 trees the preferred model was OU. We therefore used this model for all subsequent analyses. Subfamilies vary in age. To assess whether our results were driven by the age of subfamilies, we analysed the relationship between stem or crown clade ages and the residuals from the model.

The flying capacity of bats means that their biogeography is fundamentally different from nonvolant mammals. For instance, this is seen on Madagascar where nearly all mammalian clades are endemic at the subfamily level. The bats of Madagascar, on the other hand, while often endemic at the species level, are essentially never endemic at the subfamily level. The only exception is the tiny family Myzopodidae, with two extant species on Madagascar but with a fossil record in Africa as well (Gunnell, Simmons, & Seiffert, 2014). To assess the influence of bats on the results, we performed an additional set of all analyses excluding this order.

Historical contingencies could mean that clades originating in different continents have a different potential for dispersal. In particular, movement over land between Eurasia and either Africa and North America has been possible at multiple time points throughout the Neogene and Quaternary (the last 23 Myr), whereas movement between North and South America has been continuously possible as a landbridge was established between those continents, at the earliest in the Miocene (Montes et al., 2015). In contrast, land bridges have not connected the Caribbean, Madagascar or Australia with Eurasia, Africa or any of the Americas at any point during the Quaternary or Neogene. These considerations suggest that clades from the Caribbean, Madagascar, and Australia could have smaller ranges than expected. We therefore performed an additional analysis of the data excluding any subfamilies endemic to those regions.

### 2.4 Randomization

We conducted three separate randomization procedures in order to better understand the structure and signal within the data. In the first randomization procedure, we assessed whether the patterns obtained were truly driven by the subfamily‐wide traits, rather than correlations between traits in individual species. For this we randomly reassigned species to subfamilies 1,000 times while keeping the subfamily size constant and reran all analyses. Prior to the randomization, we assigned the mean value of the subfamily to any predictor variables that were missing in particular species. The adaptability traits we use cannot be calculated at the species level but only on the subfamily level. For these we instead assigned the subfamily value to all species in the subfamily before the randomization. After the randomization, we analysed the mean value for the species assigned to each subfamily in the randomization in the PGLS analyses.

In a few cases, we ended up only assigning species recently described without range information to randomized subfamilies; for those analyses, we assigned these randomized subfamilies an arbitrary range size of 1 cell. As a measure of significance for these randomizations, we used the fraction of simulations producing more extreme estimates of the effects than the empirical data. Two other procedures were designed to assess if the patterns obtained were mainly driven by a small subset of species, and are discussed in the supplementary materials (section: Randomization procedures).

### 2.5 Evolutionary success

In order to make our results of ecological success directly comparable with analyses of evolutionary success in mammalian clades, we conducted individual FiSSE analyses (Rabosky & Goldberg, 2017) of the five traits that are definable at a species level (body size, temperature niche width, precipitation niche width, annual temperature and annual precipitation; See supplementary materials section: FiSSE analyses for details). This should ensure that any differences in our conclusions compared to previous studies in terms of evolutionary success are not driven by minor methodological differences, such as our choice to focus on quantiles for niche width, as compared to the entire range as chosen in a previous study on diversification and niche width (Rolland & Salamin, 2016).

## 3 RESULTS

All the analysed predictors showed phylogenetic signal, although to varying degrees. This ranged from a relatively weak signal for precipitation niche width, with ΔAIC (relative to a white noise model) = 3.74, λ = 0.21, to a very strong phylogenetic signal for body size, with Δ AIC = 221.91, λ = 0.99 (Table 2). There was a very weak correlation between stem age and residuals, which could indicate a small effect of the subfamily age differences on the total range size of the subfamilies (*R* ^2^ = 0.03; Figure S1). This effect was absent when considering crown ages instead (*R* ^2^ < 0.01; Figure S1). A word of caution is however needed before fully dismissing a clade age effect, because crown ages are only definable for the subfamilies containing more than one species.

**Table 2:** Phylogenetic signal of each potential response variable. Median λ is the median value of Pagel's λ across the 1,000 trees, whereas median Δ AIC is the median difference in AIC for fitting Pagel's λ relative to a white noise model (i.e., a model without any phylogenetic signal)

Predictor	Median λ	Median Δ AIC
Body size	0.99	221.91
Annual temperature	0.72	13.74
Annual precipitation	0.40	7.02
Temperature niche width	0.65	31.45
Precipitation niche width	0.21	3.74
Rate of body size evolution	0.42	15.23
Rate of temperature preference evolution	0.45	5.91
Rate of precipitation preference evolution	0.92	33.89

**Figure 2:**
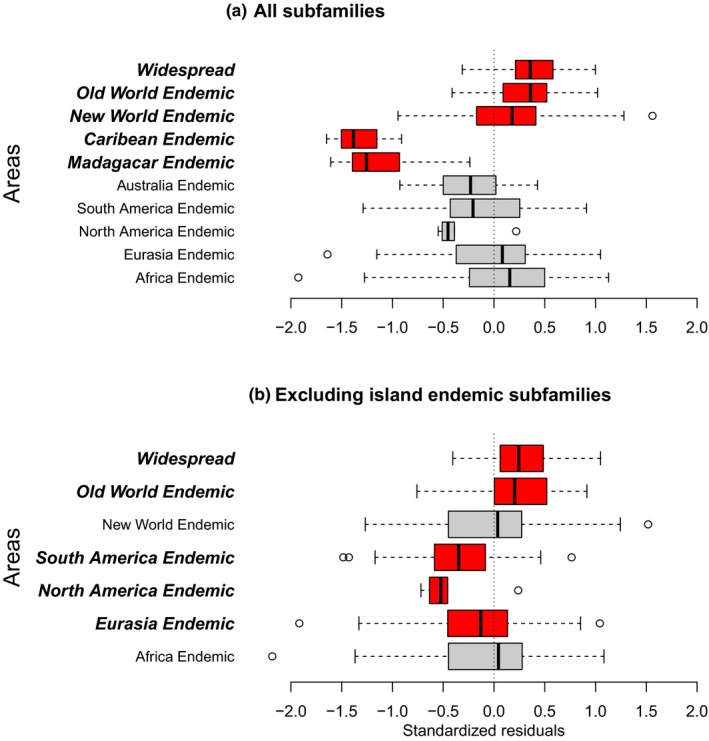
Geographical pattern in residuals. Residuals from the PGLS analyses broken down by the geographical distribution of the clades in question. (a) residuals for all subfamilies. (b) residuals for the analysis excluding subfamilies restricted to either of the island regions (Australia, Caribbean islands or Madagascar). The lower bars show subfamilies endemic to one of seven major regions (Africa, Eurasia, North America, South America, Australia, Madagascar, or Caribbean islands); the uppermost bars show the subfamilies occurring in multiple regions in the New World (North America, South America, and Caribbean islands), the Old World (remaining regions), or both the New and Old World (Widespread). The limit of the boxes are the upper and lower quartiles, with the median shown with a thick line. Whiskers extend to the median of the distribution in question plus/minus 1.5 times the inter‐quartile‐range and any outliers outside this are shown as circles. Significant deviations from zero (based on nonphylogenetic regressions) are highlighted by printing names in bold and italics [Colour figure can be viewed at wileyonlinelibrary.com]

Based on the complete dataset, we found negative residuals (i.e., smaller range sizes than predicted by the model based on nonphylogenetic regressions of residuals as a function of region) for lineages endemic to any of the island regions (Madagascar, Caribbean, and Australia), although not significantly so for Australia. We also found significantly positive residuals for lineages occurring in multiple regions (Figure 2a). For the analysis excluding the subfamilies endemic to island regions, we found significantly negative residuals for clades endemic to Eurasia and in particular clades endemic to either North or South America, and significantly positive residuals for clades occurring on both Eurasia and Africa or in both the New and Old world (Figure 2b).

**Figure 3:**
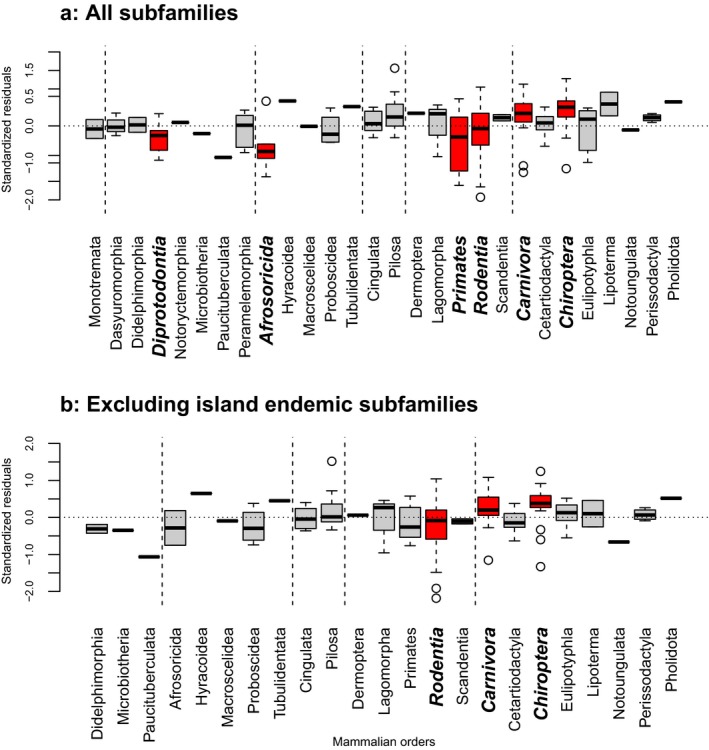
Taxonomic pattern in residuals. Residuals from the PGLS analyses broken down by order. Orders are sorted by major phylogenetic groupings (Monotremata, Marsupialia, Afrotheria, Xenarthra, Euarchontoglires, and Laurasiatheria) and within each of these alphabetically. (a) The pattern for all subfamilies. (b) The pattern excluding subfamilies restricted to either of the island regions (Australia, Caribbean islands, or Madagascar). Stippled lines separate the major groupings. The limit of the boxes are the upper and lower quartiles, with the median shown with a thick line. Whiskers extend to median plus/minus 1.5 times the inter‐quartile‐range and any outliers outside this are shown as circles. Significant deviations from zero (based on nonphylogenetic regressions) are highlighted by printing names in bold and italics [Colour figure can be viewed at wileyonlinelibrary.com]

For the analysis of the complete dataset, nonplacental mammals generally had negative residuals, although only significantly so for the mainly herbivorous Diprotodontia. Among placental mammals, significant negative residuals were found for Afrosoricida (e.g., Malagasy tenrecs), primates and rodents, whereas significantly positive residuals were found for carnivores and bats (Figure 3a). When the island endemic subfamilies are removed, Afrosoricida and primates no longer have significantly negative residuals, indicating that the pattern was largely driven by the island endemic clades, whereas the rodents still had significant negative residuals and carnivores and bats still had significantly positive residuals (Figure 3b).

**Figure 4:**
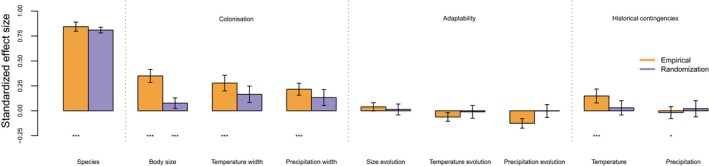
PGLS results. The standardized effect sizes and their standard errors are plotted for all potential predictors of total range size for each subfamily. Both the results using empirical data and the results of the resampling procedure are reported (see Materials and Methods). Stars are added for significance at the 0.05(*), 0.01(**), and 0.001(***) levels. For the empirical results, the *p*‐value is based on means across all 1,000 trees. For resampling, the *p*‐values are based on the fraction of simulated datasets having larger values than the empirical ones. A significant effect for the resampling means therefore that the results are unlikely to be driven by the correlation structure in the data [Colour figure can be viewed at wileyonlinelibrary.com]

As there was a very strong spatial pattern in the groups with negative residuals (i.e., drastically smaller ranges than predicted by the model) in subfamilies endemic to island regions, but the taxonomic pattern in the residuals were less marked, we focus on the results excluding the island clades in the main text. We note, however, that the results for this analysis (Figure 4) are similar to the results for the full dataset and for the dataset excluding bats (Tables S6–S8).

We found five significant positive effects of predictors associated with larger range size (listed in order of standardized effect size): number of species, body size, temperature niche width, precipitation niche width and annual temperature. We also found one negative effect (rate of precipitation preference evolution; Figure 4, Appendix S1, Table S3). Plots of the relationships between range sizes and these six predictors are presented in Figure S2, which does not show any evidence of nonlinear relationships for any of the predictors.

The effects of the rate of precipitation preference evolution and precipitation width on range size were sensitive to two of the randomization procedures, and were potentially only driven by a few species rather than representing true clade level effects (Tables S6–S8). The effect of body size was significantly stronger using the empirical data than when the species with subfamily assignments were instead randomized, indicating that the effect of body size is a true clade‐level pattern. The effects of the remaining predictors were also weaker in the randomization procedure, but not significantly so.

**Figure 5:**
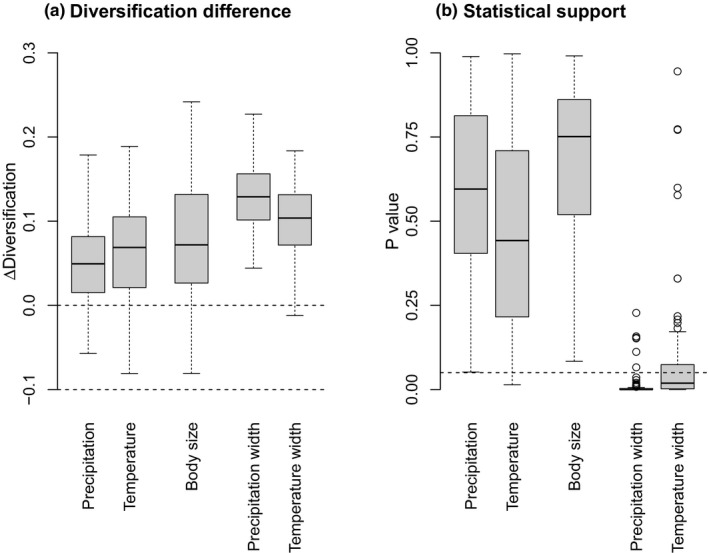
Evolutionary success. Results of FiSSE analyses estimating the importance of each of five potential factors (annual precipitation, annual temperature, body size, precipitation niche with and temperature niche width) on net diversification rates of mammals. For all analyses species are grouped into two equal sized groups based on having values of the analysed factor higher or lower than the median value and the analyses focussed on differences in diversification rates between species from these two groups. (a) Difference in diversification rate for species from dry or cold regions, small species or species with narrow niche widths relative to the remaining species across 100 trees. (b) Significance of the difference for each tree. A median value below the stippled line at *p* = 0.05 suggests that the relationship is significant for the majority of trees. Only the results for the analyses excluding island endemic clades are shown in the main figure, but results are overall similar to the analyses of other datasets (Figure S3). The limit of the boxes are the upper and lower quartiles, with the median shown with a thick line. Whiskers extend to the median of the distribution in question plus/minus 1.5 times the inter quartile range; outliers are shown as circles

On average we found higher diversification rates for species in colder and dryer regions, smaller species and species with narrower niche width (Figure 5 and Figure S3). For the analysis excluding island endemic clades, the effect sizes were larger for the two niche width traits, and the differences were significant for the majority of the tested trees (but rarely, if ever, for any of the other three traits). The results from the diversification analysis of all mammals were very similar to the analysis excluding island clades (Figure S3a,b vs Figure S3e,f). The results were, however, slightly different for the analysis excluding bats (Figure S3c,d) where the median estimated effect size was substantially larger for body size than any other trait, although only significant in around a quarter of the trees. In contrast, the effect of temperature niche width was weaker than in the other analysis and also only significant in around a quarter of the trees.

## 4 DISCUSSION

Our analysis provides strong support for colonization ability as a key factor determining ecological success, as defined by geographical distribution among mammalian clades. In particular, we found support for body size followed by niche width, in determining range sizes. Historical contingencies, both through an effect of annual temperature and, more clearly, in the spatial pattern in the model residuals, also played a role in ecological success. As the support for historical contingencies and colonization ability is evident from different analyses, we cannot reliably quantify the relative contribution of each, more than identifying a detectable/substantial role. In contrast, we found no support for larger range sizes for clades with high adaptability.

### 4.1 The importance of colonization ability

The high support for traits linked to colonization ability being related to clade total range size indicates that most mammalian clades are limited by dispersal. Support for this interpretation can also be found in the frequent occurrence of distantly related clades occupying similar niches in different regions. If there were no dispersal limitation, we should see either the consistent dominance of one clade or alternatively the frequent coexistence of clades across the world. One of the clearest examples of geographically separated but ecologically similar clades may be seen in the true moles (Eulipotyphla: Talpidae) in the Holarctic, the golden moles (Afrosoricida: Chrysochloridae) in Southern Africa and the marsupial moles (Notoryctemorphia: Notoryctidae) in Australia (IUCN, 2015; Rose & Emry, 1983). Similar support for widespread dispersal limitations among mammalian clades can be seen in examples from invasion biology, where the originally Holarctic beavers (Rodentia: Castoridae) and Palaearctic pigs (Cetartiodactyla: Suidae) are abundant in South America, whereas the originally South American coypu (Rodentia: Myocastoridae) now is abundant throughout the Holarctic (IUCN, 2015).

The strongest supported predictor related to colonization ability was body size. We note, however, that the effect of body size may be partially driven by the relationship between body size and predation. For instance, clades with a larger average body size among the mammals inhabiting South America prior to the Great American Biotic Interchange did better than clades with a smaller average body size after contact with North American competitors and predators (Faurby & Svenning, 2016a,b). Some of the larger South American animals, such as several lineages of ground sloths (Pilosa: Folivora) and to a lesser extent the glyptodonts (Cingulata: Glyptodontidae), successfully colonized much of North America, and thereby reached a large total range size. However, this was likely caused both by the global correlation between colonization ability and body size, but also by a region‐specific pattern where predation from placental carnivores may have impeded smaller mammals, but not those species large enough to be effectively safe from nonhuman predation (see discussion in Faurby & Svenning, 2016a,b).

As expected, we also recovered strong support for the role of species niche width in determining ecological success. This was clearest for temperature niche, given that the nonsignificant difference between the empirical and randomized results mean that our interpretation of the results as a clade‐wide pattern rely on the measurement of phylogenetic signal in the trait. This evidence is much stronger for temperature niche (λ = 0.65; ΔAIC = 31.45) than for precipitation niche width (λ = 0.21; ΔAIC = 3.74).

Finally, the taxonomic patterns in the residuals are also indicative of the importance of colonization success. This is most evident for bats that have a higher colonization ability than other mammals, because of their ability to fly and likely therefore have significantly larger ranges than predicted by the model (Figure 3). Similarly, the significant positive residuals of the mainly carnivorous Carnivora, and the negative residuals of the almost exclusively herbivorous Diprotodontia and the mainly herbivorous Rodentia, may together also illustrate an effect of colonization ability. This would be mediated through diet, as mammalian carnivores generally have larger home ranges than do herbivores (Kelt & Van Duren, 2001 and, therefore, potentially greater average colonization ability.

### 4.2 Historical contingencies

The primary focus of our analyses was on colonization ability versus adaptability, but our results also illustrate the importance of more idiosyncratic historical contingencies. This is evident in the significant effect of annual temperature, suggesting that warm‐adapted lineages have larger distributions. This effect may however be heavily dependent on the taxonomic level analysed. We could expect the opposite result if we were analysing sufficiently shallow taxonomic scales, at least if the debated Rapoport's rule is valid (see discussion in Pintor, Schwarzkopf, & Krockenber, 2015).

The effects of historical contingencies are also evident in the spatial pattern in the model residuals (Figure 2). There is a clear pattern between the area of regions and the range size of clades endemic to them. In the full analysis, the average range size is smallest for clades endemic to the smallest regions (Madagascar and the Caribbean islands) and also lower than expected for clades endemic to the small continent of Australia. In the dataset excluding the island endemic subfamilies (Figure 2b) it is further evident that clades endemic to the two smaller continents (North America and South America) have smaller ranges than clades endemic to either Africa or Eurasia, and that clades restricted to the New World have smaller ranges than clades restricted to the Old World. This interpretation does not, however, incorporate fossil ranges. Several clades are currently missing from other continents not because they could not reach them, but because they have gone regionally extinct. In addition to the already mentioned Myzopodidae, which went extinct in Africa but persist in Madigascar, racoons (Procyonidae) for instance had been restricted to the New World for several million years before recent translocations, although the family originated in Eurasia (Baskin, 2004).

### 4.3 No importance of adaptability?

The complete lack of support for factors related to adaptability is surprising. It suggests that there is no pattern of more adaptable clades having higher ecological success, or that we were not able to detect it under our approach. The fossil record shows that, under special circumstances, speciation can be linked to drastic changes in body size in relatively short time periods, but that such periods are rare and interspaced by long periods of apparent stasis (Uyeda, Hansen, Arnold, & Pienaar, 2011). The largest size changes are often confined to relatively unusual habitats, as for instance, in the order Proboscidae (elephants and relatives) where there are only moderate differences (in log space) in size between all the species that used to occur on continental settings, but extremely small species rapidly evolved independently on different islands (Faurby & Svenning, 2016a). It seems therefore possible that for nearly all mammalian clades, the maximum rate of body size change is not limiting their ability to adapt. This could in turn explain the lack of connection between rate of body size evolution and ecological success inferred here. If adaptability is important but just not detected here, it would likely be related to biotic interactions or adaptations to the environment, rather than to body size.

Given that we did not recover a positive relationship between the rate of niche evolution and geographical range, this could indicate that niche adaptability is unimportant for ecological success (at the level of subfamilies). We must, however, caution that this conclusion might derive from the focus on the realized rather than the fundamental niche, as the fundamental niche of most species remains unknown. The two niche spaces may, however, be drastically different, especially as our results show that mammals are dispersal limited. Some scenarios, such as peripheral speciation from a generalist ancestor, could look like extremely rapid niche evolution. This could occur because we have only focused on the differences in median climate of species. The niche of the smallest ranging species may be entirely confined within the niche of a more widely ranging one and no actual niche evolution may be taking place.

### 4.4 The contrast between ecological and evolutionary success

Returning to our initial discussion of the differences between evolutionary and ecological success, our findings that colonization factors are the primary regulators of range sizes suggest a decoupling between these different forms of clade success. This is particularly evident for niche width. We found support that larger niche width leads to higher ecological success, whereas species with small niche widths showed higher diversification rates (a result also documented by Rolland & Salamin, 2016). In addition, the strong support for higher ecological success among larger bodied clades contrasts with the relationship between this trait and evolutionary success, where our results suggest results suggest at most a weak relationship, with higher diversification rates for small species. We found moderate support for an effect of body size upon the exclusion of bats, likely because of the specific exclusion of flying foxes (Pteropodidae), which are larger than the median size of mammals, but are diversifying rapidly. There is no mechanistic argument why a potential effect of body size on diversification should not apply to bats, and we therefore see our combined results as suggesting a weak relationship between diversification rate and body size, where one of the clearest exceptions just happens to be a clade of bats. This weak pattern resembles that in earlier works, which generally found at most a weak relationship between body size and diversification rate (Gardezi & De Silva, 1999; Tomiya, 2013 and references therein).

In summary, our study shows that dispersal ability, rather than adaptability or historical contingencies, has been the major factor shaping the geographical range of mammalian clades. Future research could investigate different definitions of clade success and their underlying mechanisms, to infer differences in ecological success across the tree of life.

## BIOSKETCHES

**Søren Faurby** is a biogeographer interested in macro‐scale patterns in both ecology and evolution. Much of his recent work has been on mammals but he has conducted research on a wide variety of taxa.

**Alexandre Antonelli** is an evolutionary biologist working primarily on comparative biogeography, that is, cross‐taxonomic analyses at various taxonomic and temporal levels, combining neontological and palaeontological data, and developing analytical methods. Most of his research has dealt with the American tropics, in particular Amazonia and the Andes. Read more at http://antonelli-lab.net
