# Supplementary figures and images for: Evolutionary and ecological success is decoupled in mammals
Source: J Biogeogr. 2018 Jul 31;45(10):2227–37. doi: 10.1111/jbi.13411 (PMC6559154; doi:10.1111/jbi.13411)

**a, Stem age**

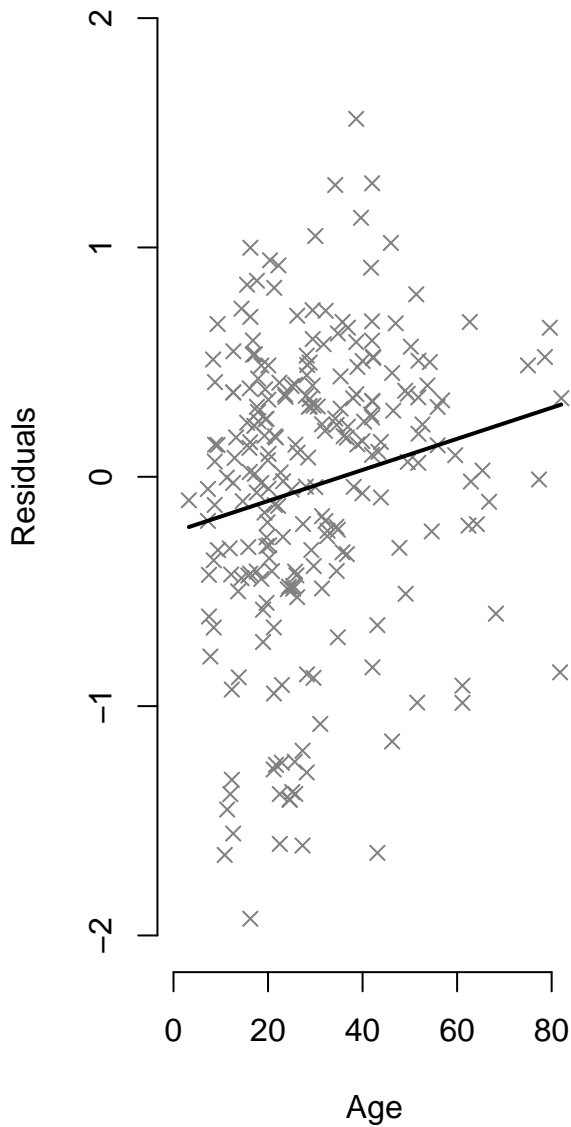

**b, Crown age**

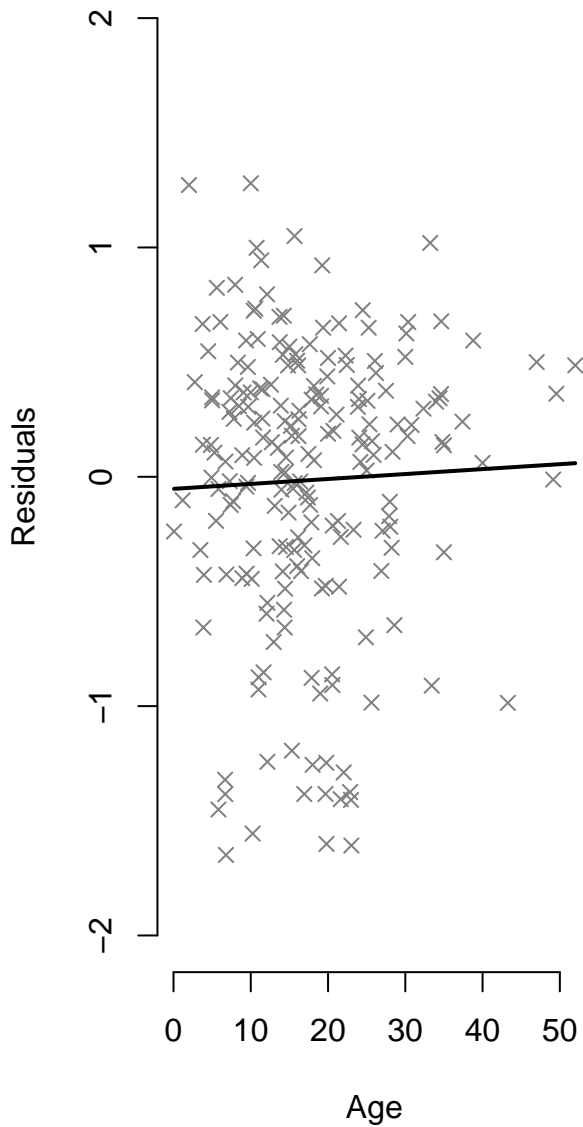

Supplement: Supplementary file 1 [file JBI-45-2227-s001.pdf]
