# Supplementary material for: Evolutionary and ecological success is decoupled in mammals
Source: J Biogeogr. 2018 Jul 31;45(10):2227–37. doi: 10.1111/jbi.13411 (PMC6559154; doi:10.1111/jbi.13411)
Supplement: Supplementary file 2 [file JBI-45-2227-s002.pdf]

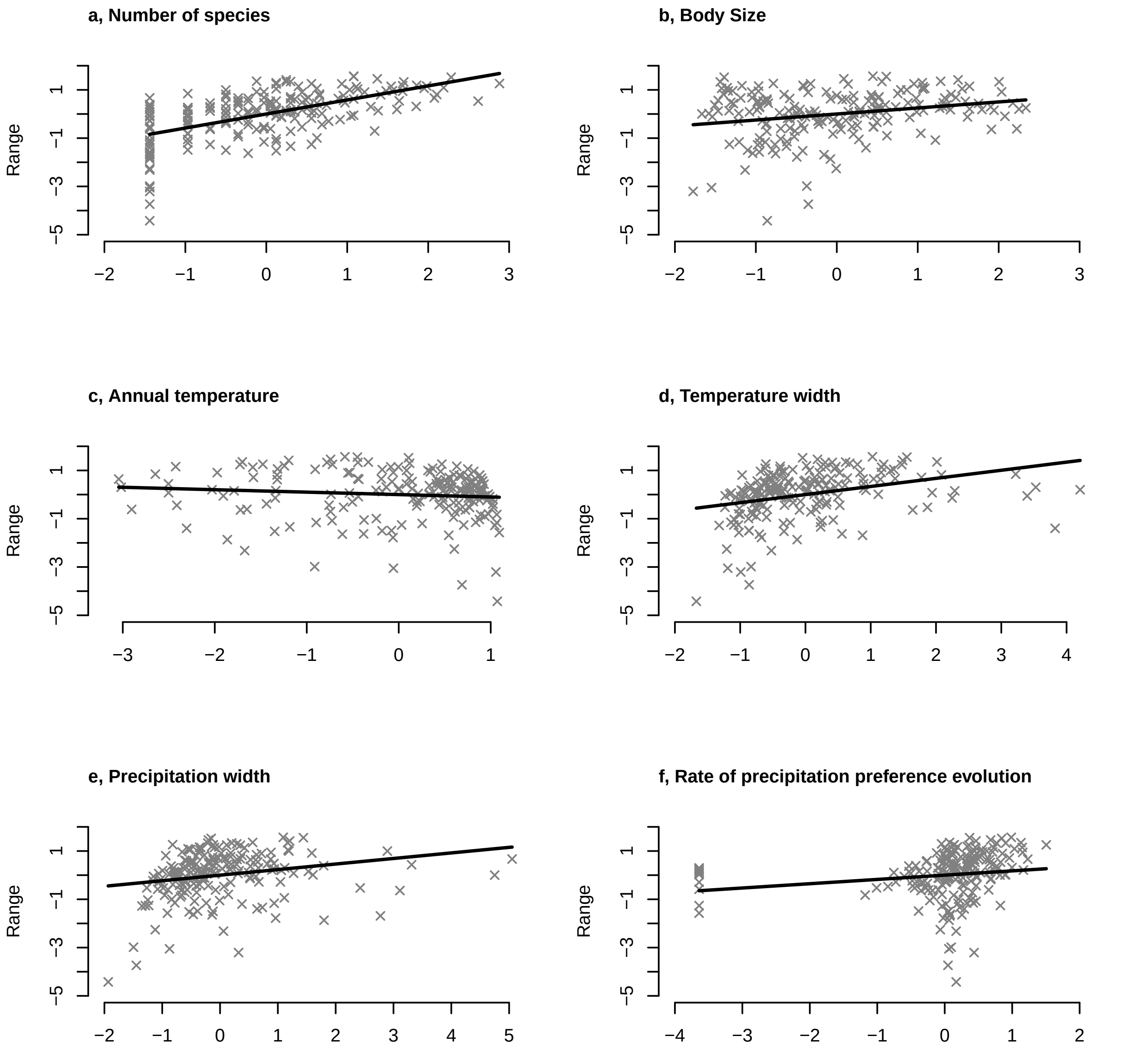

a, Number of species
b, Body Size
c, Annual temperature
d, Temperature width
e, Precipitation width
f, Rate of precipitation preference evolution
Range
