# Supplementary material for: Evolutionary and ecological success is decoupled in mammals
Source: J Biogeogr. 2018 Jul 31;45(10):2227–37. doi: 10.1111/jbi.13411 (PMC6559154; doi:10.1111/jbi.13411)
Supplement: Supplementary file 3 [file JBI-45-2227-s003.pdf]

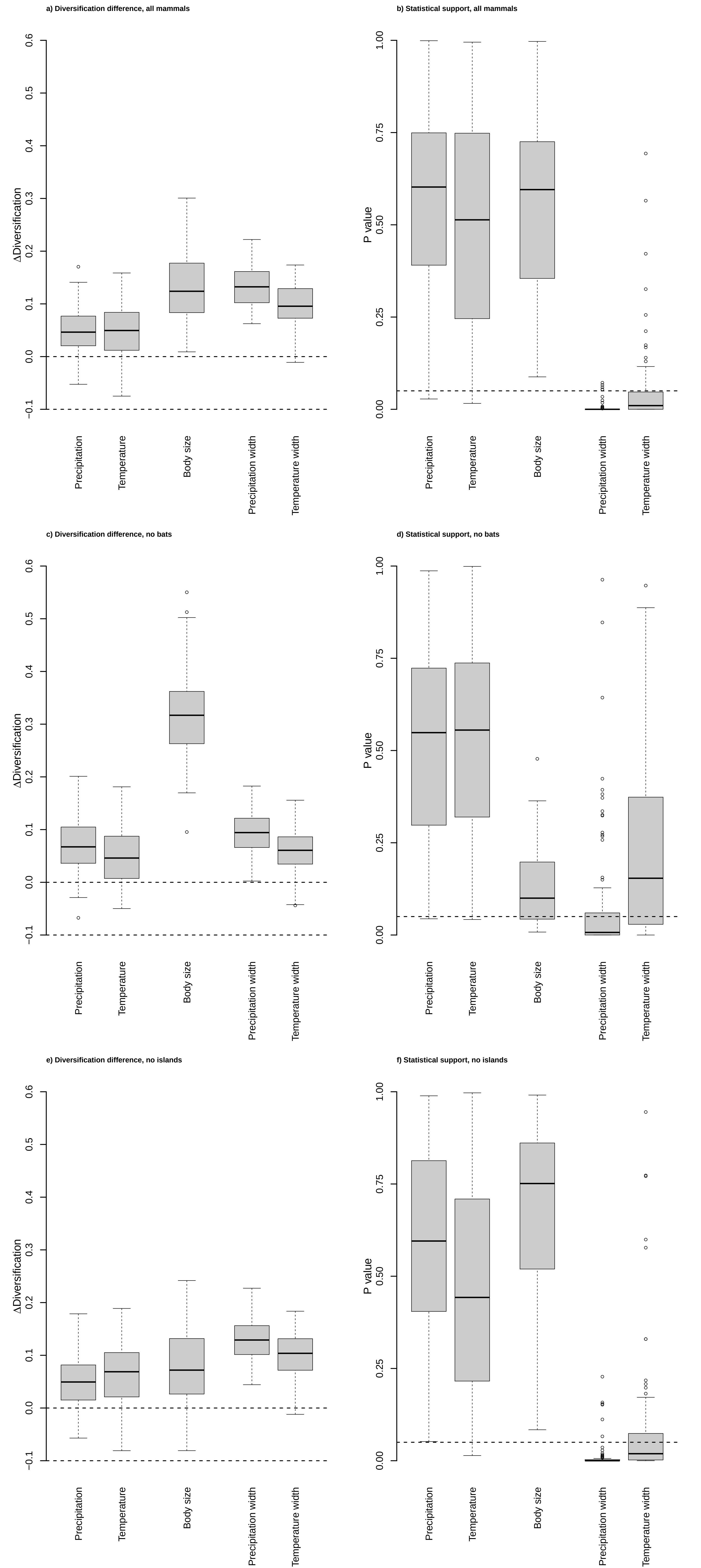
a) Diversification difference, all mammals
b) Statistical support, all mammals
c) Diversification difference, no bats
d) Statistical support, no bats
e) Diversification difference, no islands
f) Statistical support, no islands
ΔDiversification
P value
−0.1
0.0
0.1
0.2
0.3
0.4
0.5
0.6
0.00
0.25
0.50
0.75
1.00
Precipitation
Temperature
Body size
Precipitation width
Temperature width
