# Supplementary material for: Evolutionary and ecological success is decoupled in mammals
Source: J Biogeogr. 2018 Jul 31;45(10):2227–37. doi: 10.1111/jbi.13411 (PMC6559154; doi:10.1111/jbi.13411)

*Journal of Biogeography*

**SUPPORTING INFORMATION**

**Evolutionary and ecological success is decoupled in mammals**

Søren Faurby, Alexandre Antonelli

**Additional methods.**

**Taxonomy**

Analyses were conducted at the subfamily level with some subfamilies modified to ensure monophyly. Our starting point was 244 subfamilies but two were excluded for lack of body size data. By following the subfamily level we obtained a wide variation in range sizes, because some subfamilies are nearly cosmopolitan whereas others have restricted ranges. Furthermore, all extant and recently extinct species can be reliably assigned to subfamilies, whereas assignment to lower ranks such as tribes would have been problematic for lesser studied groups of mammals, such as shrews (Soricidae). For the assignment of species to subfamilies, we started with the subfamily and family assignment of Wilson & Reeder’s *Mammals of the World* (2005). We then tested the monophyly of all subfamilies based on Faurby & Svenning (2015b) and to the maximum possible extent kept the ones that were phylogenetically supported (Bayesian posterior probability of at least 0.50). We only deviated from this taxonomy for some families for which there was competing evidence available (see Table S1 in Appendix S1). We excluded marine clades (whales, pinnipeds and sirenians), because the factors underlying their geographic range could be very different to those for terrestrial mammals.

**Quantile justification**

For any mathematical distribution, the maximum value will be dependent on sample size (i.e the larger the sample size, the larger the probability that a unusually high value is included). Even if there would be no mechanistic relationship between range size and niche width, if niche width was estimated based on the most extreme values this could lead to a spurious result. Using quantiles, on the other hand, should remove these problems and be sample size independent. However, the choice of an unbiased estimator of quantiles is not straightforward, and numerous alternatives quantile estimators exist (Hyndman & Fan, 1996). In order to identify a good quantile estimator and to illustrate the potential issue of relying on maximum values instead of quantiles, we simulated random data under either a normal or a uniform distribution for the nine different estimation algorithms implemented in R. The results (Table S2) show that all nine implementations were less biased than using maximum values, but also that none of them were completely unbiased irrespective of the distribution. For our analysis we use the type 8 estimator, which is also the one recommended by Hyndman & Fan (1996), even though it is not the default in R. This has the second lowest bias for uniform distributions according to our simulations, but more importantly for our case, it has a minute negative bias under normal distributions and appears to very marginally overestimate quantiles for small sample sizes. This estimator is therefore a slightly conservative measure in our case and guarantees that we do not recover a spurious correlation between range size and niche width.

**Parameter estimation**

As a measure of body size for each subfamily, we used the mean of log-transformed weights for all species within the subfamily. These values are based on data from Faurby & Svenning (2016A), which contain the mean weight of 97% of all mammal species (5467 out of the 5633 non-marine species in our taxonomy), but does not include any information on intraspecific variation in body size. For two subfamilies (*Pachyarmaterium*: Cingulata *Insertae sedis* and Cistugidae: Chiroptera) no body-size data were available for any species and these taxa were therefore excluded from all analyses.

We identified the preferred temperature and precipitation based on the median values for all species within each subfamily. For each species we extracted annual mean temperature (Hijmans *et al.*, 2005) and log-transformed annual precipitation (Hijmans *et al.*, 2005) for each cell containing the species, and computed the median of these values for each species using Worldclim v 1.4. Finally, we calculated the mean of all species. For these traits as well as all others, we used the median value for each species, because we wanted to remove effects of outlier data. In contrast, we used the means for the subfamily data, in order to allow all species to influence the final estimate for the subfamily. Recently described species lacking range information in IUCN were omitted for the estimation of all the traits based on temperature and precipitation. We acknowledge that by solely identifying climatic preference based on contemporary climate, we will get imprecise measurements for any species whose current range limits are determined by non-climatic barriers like oceans, rivers or mountain chains. We, however, consider it likely that this should be a source of noise rather than a systematic bias and therefore that it is unlikely to influence our conclusions.

We identified niche widths based on the difference between the 25% and 75% quantiles of either annual mean temperature or log-transformed annual precipitation of all cells occupied by each species. We again used the mean of the niche widths estimated for each species within the subfamily. We acknowledge that there are numerous niche dimensions other than just temperature and precipitation, and that numerous other dimensions could be included to estimate the width of the species niches. However, many other niche dimensions would either be strongly correlated to one of our two environmental predictors (as would be the case for other climatic predictors) or be extremely difficult to assign values to the extinct and least known extant species (as would for instance be the case for the division between nocturnal or diurnal species). There was no specific justification for using the 25% and 75% quantiles over others, but we note that the lack of a correlation between quantiles and sample size is not true for very small samples and the relationship between 75% quantile is approximately uncorrelated to sample sizes for sample sizes of at least 4, whereas e.g. the 90% requires a minimum sample size of 10 for this to be true.

We identified rates of evolution of body size, median annual temperature and median log annual precipitation for each subfamily based on σ^2^ rates from OU models using the library OUwie (Beaulieu & O'Meara, 2014). We did this for 100 trees from Faurby et al (2015B) and used the median value across the trees in all analyses. Measurement errors were incorporated into the analyses ((log10(1.1) i.e. log10 of annual precipitation in mm for precipitation, 0.1 i.e. log10 of weight in grams for body size, and 0.5°C for annual temperature)). For subfamilies with less than five species, we added random species from the sister lineage to have five species to estimate the parameters from. Faurby et al (2015B) used birth-death simulated ages for species lacking generic data, which occasionally generated unrealistically young ages for some species. We therefore added 0.01 million years to the age of all branches to the trees prior to the calculation of rates. This value roughly corresponds to the latest plausible speciation events in mammals at the end of the last ice age (see discussion in Faurby & Svenning 2016A).

**Randomisation procedures**

In addition to the main randomization procedure described in the main text we employed two additional types of rearrangements to assess whether the results were consistent across the majority of species in each clade, or mainly driven by a smaller subset of species. We assessed this through jackknifing, creating 1000 new sets containing only two thirds of the species (across all species, i.e. not within the individual subfamily). In this procedure only the ranges were modified, whereas the predictor values for the subfamilies were kept constant. Subfamilies from which all species were removed in a given replicate were excluded from the analyses of that replicate. As a measure of significance of the jackknife results, we used the fraction of replicates having estimated effect sizes in the opposite direction to the empirical results.

In another procedure we assessed whether the results were driven by a few species with particularly large geographic ranges. In this procedure, we systematically removed between 1–50% of the species with the largest geographic range (again across all species, i.e. not within the individual subfamily). As in the previous randomisation procedure, only the ranges were modified, whereas the predictor values for the subfamilies were kept constant. Subfamilies from which all species were removed in a given replicate were excluded from the analyses of that replicate.

Including the analyses of the empirical results, and the three sets of randomizations across 1000 trees for three separate datasets we ran a total of 6,153,000 PGLS analyses. The algorithm failed to converge in 694 of these analyses (0.011%), which were subsequentely excluded. Thus, for a few of the randomisations we only combined the results from between 994 to 999 trees rather than all 1000.

All analyses for the randomisation (as well as all other steps) were conducted in R (R Core Team 2017) using the libraries adephylo (Jombart & Dray, 2010), ape (Paradis *et al.,* 2004), diversitree (Fitzjohn, 2012), geiger (Harmon *et al.,* 2008), OUwie (Beaulieu & O'Meara, 2014), phangorn (Schliep, 2011), phylobase (R Hackathon *et al.*, 2017), phylolm (Ho & Ané, 2014), phytools (Revell, 2012) and raster (Hijmans & van Etten, 2012).

**FiSSE analyses**

Trait dependent diversification has often been analysed thorough BiSSE models (Maddison *et al.* 2007) or a suite of derived models. These models have however been found to show a very high false positive rate, which is likely caused by improper null models. To analyse trait depended diversification we therefore used the newly developed FiSSE model, which has been found through simulations to have a better statistical behaviour than its predecessors (Rabosky & Goldberg 2017). We did however make two changes relative to the initial suggestion of Rabosky & Goldberg (2017):

1. Due to the shape of our empirical trees there was occasionally a big difference between the empirical number of trait changes on the trees and the number of changes for simulated traits. We therefore had to increase the tolerance for the deviation between the two from 10% as used in the simulations of Rabosky & Goldberg (2017) to 50%. Simulations conducted by Rabosky & Goldberg (2017) suggest that the statistical behaviour of the model is little affected by changes in tolerance level, meaning that it is unlikely to have a major effect for our conclusions;
2. Also due to the shape of the empirical trees we modified the codes to use the median diversification rate for species with each trait value, rather than the mean of these, as used in the original FiSSE implementation. Medians are generally a better descriptor of the central tendency of a distribution for non-symmetrical distributions. In our particular case we note that the mean diversification rates across species were around twice as large as the medians (results not shows), which highlights that the means were driven by a limited number of values. There is no a priori reason why means should always be better than medians for FiSSE analyses (Dan Rabosky and Emma Goldberg, pers. com.) and in our case we therefore believe that our choice of medians better describe the underlying pattern in the tree. We note however that since means have a smaller variance than medians and the FiSSE algorithm potentially may have a relatively small power, other researchers (especially if they are working on relatively small sized trees with only moderate variation in diversification rate between species) should consider using means as in the original implementation rather than medians as we do here.

We conducted FiSSE models (Rabosky & Goldberg, 2017) determining the importance of each of the five potential parameters (Size, Temperature, Precipitation, Precipitation niche width, Temperature niche width) on diversification rate. In order to improve comparisons with the tests of ecological success, we conducted separate analyses based on i) all non-marine mammals, ii) all species except for bats, and iii) all subfamilies except for those that are island endemic. These three sets of analyses correspond to the same ones analyzed for ecological success. Species with missing values for the trait in question were replaced with the median value within the subfamily. For all FiSSE analyses, we grouped all species into ranked groups of equal size, i.e. the smallest versus the largest half of all species. All analyses were constructed on 100 separate trees to incorporate phylogenetic uncertainty. Like in the estimation of niche evolution, and for the same reason, we added 0.01 Ma to the age of all species prior to the analyses. We estimated the effect size as the difference in diversification rate between each trait value divided by the median value across all species and the statistical support based on the fraction of the 100 trees showing significant effect sizes.

**Additional references**

Beaulieu, J. M. & O'Meara B. ( 2014). *OUwie: Analysis of evolutionary rates in an OU framework*. https://cran.r-project.org/web/packages/OUwie/

Beaulieu, J. M. & O'Meara B. (2016). Detecting hidden diversification shifts in models of trait-dependent speciation and extinction. *Systematic Biology*, 65, 583-601.

Fitzjohn, R. G. (2012). Diversitree: comparative phylogenetic analyses of diversification in R. *Method in ecology and evolution*, 3, 1084-1092.

Harmon, L. J., Weir, J. T., Brock, C. D., Glor, R. E. & Challenger, W. (2008). GEIGER: investigating evolutionary radiations. *Bioinformatics*, 24, 129-131.

Hijmans, R. J., Cameron, S. E., Parra, J. L., Jones, P. G. & Jarvis, A. (2005). Very high resolution interpolated climate surfaces for global land areas. *International Journal of Climatology*, *25*, 1965–1978.

Hijmans, R. J. & van Etten J. (2012)*. Raster: Geographic analysis and modeling with raster data. R package version 2.0-12*. https://CRAN.R-project.org/package=raster

Ho, L. S. T. & Ané, C. (2014). A linear-time algorithm for Gaussian and non-Gaussian trait evolution models. *Systematic Biology*, 63, 397–408.

Hyndman, R.J., & Fan, Y. (1996) Sample quantiles in statistical packages, American Statistician 50, 361–365.

Jombart, T. Dray S. (2010). Adephylo: exploratory analyses for the phylogenetic comparative method. *Bioinformatics*, *26*, 1907-1909.

Koepfli, K.P., Deere, K.A., Slater, G.J., Begg, C., Begg, K., Grassman, L., Lucherini, M., Veron, G. & Wayne, R.K. (2008) Multigene phylogeny of the Mustelidae: resolving relationships, tempo and biogeographic history of a mammalian adaptive radiation. BMC biology, 6, 10.

Maddison W.P., Midford P.E., and Otto S.P. 2007. Estimating a binary character’s effect on speciation and extinction. *Systematic Biology*, *56*, 701-710

Paradis E., Claude J. & Strimmer K. (2004). APE: analyses of phylogenetics and evolution in R language. *Bioinformatics*, *20*, 289-290.

R Core Team (2017). *R: A language and environment for statistical computing. R Foundation for Statistical Computing, Vienna, Austria*. https://www.R-project.org/.

R Hackathon *et al*. (2017). *Phylobase: Base Package for Phylogenetic Structures and Comparative Data. R package version 0.8.4*. https://CRAN.R-project.org/package=phylobase

Revell, L. J. (2012). Phytools: An R package for phylogenetic comparative biology (and other things). *Methods in Ecology and Evolution*, *3*, 217-223.

Schliep, K. P. (2011). phangorn: phylogenetic analysis in R. *Bioinformatics*, *27*, 592-593.

Wilson, D. E. & Reeder D. M. (2005). *Mammal Species of the World. A Taxonomic and Geographic Reference (3rd ed),* Johns Hopkins University Press

**Appendix S1: Supplementary tables**

| **Table S1: Deviations from the taxonomy of Wilson & Reeder (2005) .** | | |
| --- | --- | --- |
| Order | Family | Deviation |
| Afrosoricida | Chrysochloridae | All subfamilies are merged since neither of the classical subfamilies (Chrysochlorinae are Amblysominae) is monophyletic in Faurby & Svenning (2015B). |
| Carnivora | Eupleridae | All subfamilies are merged since one of the classical subfamilies (Euplerinae) is not monophyletic in Faurby & Svenning (2015B). |
|  | Felidae | We merged the subfamilies Felinae and Pantherinae because even though they are both monophyletic, their split is only placed in the Late Miocene. We therefore judged it to be more consistent with the taxonomy of the other families to merge them. |
|  | Mustelidae | We followed the more recent taxonomy of Koepfli *et al.* (2008) which is based on recent phylogenetic analyses of the family. |
|  | Ursidae | We split the extant species into the three classical subfamilies that Wilson & Reeder (2005) discuss but ultimately chose not to accept. |
|  | Viverridae | *Macrogalidia musschenbroekii* assigned to Hemigalinae rather than Paradoxurinae. |
| Cetartiodactyla | Bovidae | The taxonomy of the family is inconsistent as many of the clades considered tribes by Wilson & Reeder have been considered subfamilies by other sources. We therefore analysed these clades as if they were subfamilies. We classified *Oreotragus* as a separate clade containing this single genus, since the genus was not closely related to the normally assigned Neotragini in the phylogeny of Faurby & Svenning (2015B). |
|  | Cervidae | *Hydropotes* is nested within Capreolinae in Faurby and Svenning (2015B) and Hydropotinae and Capreolinae are therefore merged and called Capreolinae. |
| Chiroptera | Molossidae | Subfamilies merged since they are not monophyletic in Faurby and Svenning (2015B) |
|  | Phyllostomidae | Subfamilies merged since most are not monophyletic in Faurby and Svenning (2015B) |
|  | Vespertilionidae | Antrozoinae and *Lasionycteris noctivagans* classified as Vespertilioninae based on Faurby and Svenning (2015B) |
| Cingulata | Dasypodidae | Chlamyphorinae accepted as a separate subfamily distinct from Euphractinae |
| Diprotodontia | Phalangeridae | All subfamilies are merged since one of the classical subfamilies (Phalangerinae) is not monophyletic in Faurby and Svenning (2015B). |
|  | Pseudocheiridae | All subfamilies are merged since two of the classical subfamilies (Pseudocheirinae and Pseudochiropsinae) are not monophyletic in Faurby and Svenning (2015B). |
| Eulipotyphla | Talpidae | All subfamilies are merged since two of the classical subfamilies (Scalopinae and Talpinae) are not monophyletic in Faurby & Svenning (2015B). |
| Rodentia | Anomaluridae | All subfamilies are merged since Zenkerellinae is not supported in Faurby & Svenning (2015B). |
|  | Dipodidae | All subfamilies are merged since two of the classical subfamilies (Dipodinae and Allactaginae) are not monophyletic in Faurby & Svenning (2015B). |
|  | Echimyidae | All subfamilies are merged since two of the classical subfamilies (Echimyinae and Eumysopinae) are not monophyletic in Faurby & Svenning (2015B). |
|  | Muridae | Otomyinae is nested within Murinae in Faurby & Svenning (2015B) and Otomyinae and Murinae are therefore merged and called Murinae. |
|  | Nesomyidae | Cricetomyinae is paraphyletic in Faurby & Svenning (2015B) and is therefore split into Saccostomurinae (*Saccostomus*) and Cricetomyinae (*Beamys* and *Cricetomys*) that previously had been considered subfamilies. |
|  | Spalacidae | Tachyoryctinae grouped with Rhizomyinae since Tachyoryctinae is nested within Rhizomyinae in Faurby & Svenning (2015B) |
|  | | |

| **Table S2**  Relationship between sample size and 75% quantile based on the nine different types implemented in R for various sample sizes (N). The median value across 10,000 randomizations for five different sizes of randomly generated vectors are given along with the estimated slope between size of the vector and estimate assuming a linear model. For comparison, the same is given for the maximum value of the distributions. | | | | | | |
| --- | --- | --- | --- | --- | --- | --- |
| **Test 1**. Normal distribution, mean=0, standard deviation =1  (expected quantile for infinite sample size ≈ 0.675) | | | | | | |
|  | N=4 | N=8 | N=12 | N=16 | N=20 | Slope |
| Type 1 | 0.279 | 0.465 | 0.537 | 0.564 | 0.585 | 0.0176 |
| Type 2 | 0.643 | 0.656 | 0.664 | 0.660 | 0.662 | 0.0006 |
| Type 3 | 0.279 | 0.465 | 0.537 | 0.564 | 0.585 | 0.0176 |
| Type 4 | 0.279 | 0.465 | 0.537 | 0.564 | 0.585 | 0.0176 |
| Type 5 | 0.643 | 0.656 | 0.664 | 0.660 | 0.662 | 0.0006 |
| Type 6 | 0.824 | 0.750 | 0.727 | 0.706 | 0.701 | -0.0079 |
| Type 7 | 0.456 | 0.555 | 0.600 | 0.609 | 0.623 | 0.0091 |
| Type 8 | 0.688 | 0.678 | 0.680 | 0.672 | 0.671 | -0.0023 |
| Type 9 | 0.688 | 0.678 | 0.680 | 0.672 | 0.671 | -0.0016 |
| Max | 0.991 | 1.386 | 1.583 | 1.719 | 1.817 | 0.0503 |
| **Test 2**. Uniform distribution, minimum=0, maximum =1  (expected quantile for infinite sample size =0.75) | | | | | | |
| Type 1 | 0.618 | 0.678 | 0.701 | 0.715 | 0.723 | 0.0067 |
| Type 2 | 0.720 | 0.737 | 0.740 | 0.744 | 0.747 | 0.0023 |
| Type 3 | 0.618 | 0.678 | 0.701 | 0.715 | 0.723 | 0.0067 |
| Type 4 | 0.618 | 0.678 | 0.701 | 0.715 | 0.723 | 0.0067 |
| Type 5 | 0.720 | 0.737 | 0.740 | 0.744 | 0.747 | 0.0023 |
| Type 6 | 0.782 | 0.768 | 0.761 | 0.760 | 0.759 | 0.0000 |
| Type 7 | 0.666 | 0.707 | 0.721 | 0.729 | 0.734 | 0.0045 |
| Type 8 | 0.740 | 0.747 | 0.747 | 0.750 | 0.750 | 0.0015 |
| Type 9 | 0.735 | 0.745 | 0.745 | 0.749 | 0.750 | 0.0017 |
| Max | 0.842 | 0.918 | 0.944 | 0.957 | 0.966 | 0.0089 |

| **Table S3: Taxonomic and clade age information of mammalian subfamilies.** | | | | | | |  |
| --- | --- | --- | --- | --- | --- | --- | --- |
| Order | Family | Subfamily | Number of species | Median Stem Age in Ma across 1000 trees | Median Crown Age in Ma across 1000 trees | Endemic to island regions |  |
| Afrosoricida | *Insertae sedis* | Bibymalagasia | 1 | 7.8 | NA | Yes | |
|  | Chrysochloridae |  | 21 | 68.2 | 12.0 | No | |
|  | Tenrecidae | Geogalinae | 1 | 24.2 | NA | Yes | |
|  |  | Oryzorictinae | 26 | 25.1 | 22.9 | Yes | |
|  |  | Potamogalinae | 3 | 47.0 | 21.4 | No | |
|  |  | Tenrecinae | 5 | 29.6 | 17.9 | Yes | |
| Carnivora | Ailuridae | Ailurinae | 1 | 31.1 | NA | No | |
|  | Canidae | Caninae | 41 | 46.5 | 16.3 | No | |
|  | Eupleridae |  | 10 | 21.7 | 18.0 | Yes | |
|  | Felidae | Felinae | 41 | 28.0 | 9.4 | No | |
|  |  | Machairodontinae | 4 | 28.0 | 19.1 | No | |
|  | Herpestidae |  | 34 | 21.7 | 11.6 | No |  |
|  | Hyaenidae |  | 4 | 26.3 | 5.3 | No |  |
|  | Mephitidae |  | 13 | 32.1 | 20.7 | No |  |
|  | Mustelidae | Galictinae | 7 | 17.2 | 15.8 | No |  |
|  |  | Helictidinae | 4 | 19.4 | 11.5 | No |  |
|  |  | Lutrinae | 13 | 16.3 | 13.7 | No |  |
|  |  | Martinae | 10 | 20.4 | 11.4 | No |  |
|  |  | Melinae | 4 | 21.3 | 5.6 | No |  |
|  |  | Mellivorinae | 1 | 22.4 | NA | No |  |
|  |  | Mustelinae | 19 | 16.3 | 10.8 | No |  |
|  |  | Taxidiinae | 1 | 25.8 | NA | No |  |
|  | Nandiniidae |  | 1 | 39.7 | NA | No |  |
|  | Prionodontidae |  | 2 | 29.5 | 10.9 | No |  |
|  | Procyonidae |  | 14 | 28.8 | 24.0 | No |  |
|  | Ursidae | Ailuropodinae | 1 | 17.2 | NA | No |  |
|  |  | Tremarctinae | 5 | 9.3 | 4.8 | No |  |
|  |  | Ursinae | 7 | 9.3 | 3.8 | No |  |
|  | Viverridae | Hemigalinae | 5 | 20.1 | 15.45 | No |  |
|  |  | Paradoxurinae | 6 | 20.1 | 17.4 | No |  |
|  |  | Viverrinae | 22 | 23.6 | 18.5 | No |  |
| Cetartiodactyla | Antilocapridae | Antilocaprinae | 4 | 19.7 | 12.2 | No |  |
|  | Bovidae | Aepycerotini | 4 | 15.6 | 10.4 | No |  |
|  |  | Alcelaphini | 10 | 7.3 | 5.8 | No |  |
|  |  | Antilopini | 38 | 11.9 | 10.4 | No |  |
|  |  | Boselaphini | 2 | 14.6 | 7.7 | No |  |
|  |  | Bovini | 15 | 12.6 | 10.0 | No |  |
|  |  | Caprini | 43 | 8.7 | 7.4 | No |  |
|  |  | Cephalophini | 18 | 12.9 | 10.4 | No |  |
|  |  | Hippotragini | 8 | 7.3 | 5.5 | No |  |
|  |  | *Oreotragus* | 1 | 8.4 | NA | No |  |
|  |  | Reduncini | 9 | 8.7 | 6.7 | No |  |
|  |  | Tragelaphini | 9 | 12.6 | 9.6 | No |  |
|  | Camelidae |  | 10 | 65.4 | 25.0 | No |  |
|  | Cervidae | Capreolinae | 33 | 17.1 | 15.2 | No |  |
|  |  | Cervinae | 44 | 16.1 | 13.5 | No |  |
|  | Giraffidae | Giraffinae | 2 | 19.7 | 7.8 | No |  |
|  | Hippopotamidae |  | 6 | 53.7 | 8.0 | No |  |
|  | Moschidae | Moschinae | 7 | 19.7 | 13.7 | No |  |
|  | Suidae | Suinae | 21 | 28.8 | 8.3 | No |  |
|  | Tayassuidae | Tayassuinae | 6 | 28.8 | 8.5 | No |  |
|  | Tragulidae |  | 10 | 40.3 | 37.4 | No |  |
| Chiroptera | Cistugidae ^a^ |  | 2 | 41.6 | 1.3 | No |  |
|  | Craseonycteridae |  | 1 | 46.3 | NA | No |  |
|  | Emballonuridae | Emballonurinae | 34 | 42.0 | 38.8 | No |  |
|  |  | Taphozoinae | 18 | 42.0 | 34.6 | No |  |
|  | Furipteridae |  | 2 | 34.2 | 11.5 | No |  |
|  | Hipposideridae |  | 83 | 42.2 | 30.0 | No |  |
|  | Megadermatidae |  | 5 | 46.3 | 26.2 | No |  |
|  | Molossidae |  | 100 | 49.6 | 49.6 | No |  |
|  | Mormoopidae |  | 10 | 35.8 | 30.4 | No |  |
|  | Mystacinidae |  | 2 | 47.8 | 28.2 | Yes |  |
|  | Myzopodidae |  | 2 | 54.6 | 0.1 | Yes |  |
|  | Natalidae |  | 11 | 51.8 | 16.0 | No |  |
|  | Noctilionidae |  | 2 | 34.2 | 2.0 | No |  |
|  | Nycteridae |  | 16 | 51.4 | 12.1 | No |  |
|  | Phyllostomidae |  | 178 | 35.8 | 35.0 | No |  |
|  | Pteropodidae |  | 186 | 62.9 | 16.4 | No |  |
|  | Rhinolophidae |  | 74 | 42.2 | 20.0 | No |  |
|  | Rhinopomatidae |  | 4 | 52.7 | 29.0 | No |  |
|  | Thyropteridae |  | 4 | 42.0 | 10.0 | No |  |
|  | Vespertilionidae | Kerivoulinae | 25 | 29.3 | 24.5 | No |  |
|  |  | Miniopterinae | 22 | 46.0 | 33.2 | No |  |
|  |  | Murininae | 20 | 29.4 | 23.9 | No |  |
|  |  | Myotinae | 102 | 34.8 | 30.1 | No |  |
|  |  | Vespertilioninae | 242 | 38.7 | 34.6 | No |  |
| Cingulata | *Insertae sedis* | *Pachyarmaterium* ^a^ | 1 | 23.6 | NA | No |  |
|  | Dasypodidae | Chlamyphorinae | 2 | 25.0 | 14.0 | No |  |
|  |  | Dasypodinae | 10 | 40.0 | 26.0 | No |  |
|  |  | Euphractinae | 6 | 29.2 | 15.6 | No |  |
|  |  | Tolypeutinae | 7 | 25.0 | 18.2 | No |  |
|  | Glyptodontidae |  | 9 | 21.4 | 17.7 | No |  |
|  | Pampatheridae |  | 5 | 21.4 | 16.2 | No |  |
| Dasyuromorphia | Dasyuridae | Dasyurinae | 43 | 19.7 | 16.1 | Yes |  |
|  |  | Sminthopsinae | 29 | 18.6 | 15.7 | Yes |  |
|  | Myrmecobiidae |  | 1 | 23.7 | NA | Yes |  |
|  | Thylacinidae |  | 1 | 30.0 | NA | Yes |  |
| Dermoptera | Cynocephalidae |  | 2 | 82.0 | 7.4 | No |  |
| Didelphimorphia | Didelphidae | Caluromyinae | 5 | 31.4 | 25.4 | No |  |
|  |  | Didelphinae | 94 | 31.4 | 27.9 | No |  |
| Diprotodontia | Acrobatidae |  | 2 | 42.0 | 25. | Yes |  |
|  | Burramyidae |  | 5 | 43.9 | 34.8 | Yes |  |
|  | Diprotodontidae | Diprotodontinae | 1 | 8.5 | NA | Yes |  |
|  |  | Palorchestinae | 1 | 13.7 | NA | Yes |  |
|  |  | Zygomaturinae | 2 | 8.5 | 3.9 | Yes |  |
|  | Hypsiprymnodontidae |  | 2 | 32.1 | 21.3 | Yes |  |
|  | Macropodidae | Lagostrophinae | 2 | 12.2 | 6.8 | Yes |  |
|  |  | Macropodinae | 75 | 12.3 | 11.0 | Yes |  |
|  |  | Sthenurinae | 9 | 13.8 | 11.0 | Yes |  |
|  | Petauridae |  | 12 | 34.8 | 23.3 | Yes |  |
|  | Phalangeridae |  | 26 | 43.9 | 17.4 | Yes |  |
|  | Phascolarctidae |  | 1 | 32.5 | NA | Yes |  |
|  | Potoroidae |  | 12 | 22.2 | 13.1 | Yes |  |
|  | Pseudocheiridae |  | 19 | 34.8 | 24.9 | Yes |  |
|  | Tarsipedidae |  | 1 | 38.1 | NA | Yes |  |
|  | Thylacoleonidae |  | 1 | 27.4 | NA | Yes |  |
|  | Vombatidae |  | 6 | 21.2 | 14.4 | Yes |  |
| Eulipotyphla | Erinaceidae | Erinaceinae | 16 | 38.9 | 9.6 | No |  |
|  |  | Galericinae | 8 | 38.9 | 24.5 | No |  |
|  | Nesophontidae |  | 8 | 61.1 | 43.3 | Yes |  |
|  | Solenodontidae |  | 3 | 61.1 | 33.42 | Yes |  |
|  | Soricidae | Crocidurinae | 211 | 34.5 | 30.8 | No |  |
|  |  | Myosoricinae | 19 | 34.5 | 26.9 | No |  |
|  |  | Soricinae | 148 | 38.0 | 34.4 | No |  |
|  | Talpidae |  | 41 | 75.0 | 52.0 | No |  |
| Hyracoidea | Procaviidae |  | 5 | 62.7 | 6.01 | No |  |
| Lagomorpha | Leporidae |  | 63 | 50.2 | 15.0 | No |  |
|  | Ochotonidae |  | 31 | 42.1 | 34.0 | No |  |
|  | Prolagidae |  | 1 | 42.1 | NA | No |  |
| Litopterna | Macraucheniidae | Macraucheniinae | 2 | 41.8 | 21.1 | No |  |
|  | Proterotheriidae | Megadolodinae | 1 | 41.8 | NA | No |  |
| Macroscelidea | Macroscelididae |  | 19 | 77.3 | 49.1 | No |  |
| Microbiotheria | Microbiotheriidae |  | 1 | 64.2 | NA | No |  |
| Monotrremata | Ornithorhynchidae |  | 1 | 36.7 | NA | Yes |  |
|  | Tachyglossidae |  | 6 | 36.7 | 24.0 | Yes |  |
| Notoryctemorphia | Notoryctidae |  | 2 | 59.6 | 8.9 | Yes |  |
| Notoungulata | Toxodontidae | Toxodontinae | 3 | 66.8 | 28.0 | No |  |
| Paucituberculata | Caenolestidae |  | 6 | 81.8 | 11.7 | No |  |
| Peramelemorphia | Chaeropodidae |  | 1 | 17.9 | NA | Yes |  |
|  | Peramelidae | Echymiperinae | 10 | 18.9 | 14.3 | Yes |  |
|  |  | Peramelinae | 7 | 22.7 | 14.5 | Yes |  |
|  |  | Peroryctinae | 4 | 18.9 | 13.0 | Yes |  |
|  | Thylacomyidae |  | 2 | 17.9 | 7.2 | Yes |  |
| Perissodactyla | Equidae |  | 12 | 56.8 | 5.0 | No |  |
|  | Rhinocerotidae | Elasmotheriinae | 1 | 42.0 | NA | No |  |
|  |  | Rhinocerotinae | 8 | 42.0 | 25.9 | No |  |
|  | Tapiridae |  | 8 | 51.8 | 20.0 | No |  |
| Pholidota | Manidae |  | 9 | 79.6 | 25.3 | No |  |
| Pilosa | Bradypodidae |  | 4 | 22.2 | 19.2 | No |  |
|  | Cyclopedidae |  | 1 | 38.7 | NA | No |  |
|  | Megalonychidae | Megalonychinae | 13 | 18.0 | 14.4 | No |  |
|  | Megatheriidae | Megatheriinae | 3 | 8.8 | 3.8 | No |  |
|  | Mylodontidae | Lestodontinae | 2 | 3.2 | 1.2 | No |  |
|  |  | Mylodontinae | 2 | 9.4 | 3.5 | No |  |
|  |  | Scelidotheriinae | 4 | 11.3 | 4.9 | No |  |
|  | Myrmecophagidae |  | 3 | 38.7 | 13.7 | No |  |
|  | Nothrotheridae |  | 2 | 8.8 | 2.8 | No |  |
| Primates | Aotidea |  | 11 | 19.3 | 15.3 | No |  |
|  | Archaeolemuridae |  | 3 | 25.6 | 12.1 | Yes |  |
|  | Atelidae | Alouattinae | 12 | 16.1 | 11.2 | No |  |
|  |  | Atelinae | 16 | 16.1 | 14.0 | No |  |
|  | Callitrichidae |  | 41 | 19.3 | 14.9 | No |  |
|  | Cebidae | Cebinae | 9 | 16.8 | 14.1 | No |  |
|  |  | Saimiriinae | 5 | 16.8 | 9.4 | No |  |
|  | Cercopithecidae | Cercopithecinae | 67 | 17.6 | 17.2 | No |  |
|  |  | Colobinae | 56 | 17.6 | 14.1 | No |  |
|  | Cheirogaleidae |  | 32 | 24.6 | 23.0 | Yes |  |
|  | Daubentoniidae |  | 2 | 51.6 | 25.6 | Yes |  |
|  | Galagidae |  | 18 | 35.4 | 19.9 | No |  |
|  | Hominidae |  | 10 | 14.4 | 10.6 | No |  |
|  | Hylobatidae |  | 16 | 14.4 | 8.9 | No |  |
|  | Indriidae |  | 19 | 22.5 | 19.8 | Yes |  |
|  | Lemuridae |  | 23 | 27.3 | 23.0 | Yes |  |
|  | Lepilemuridae |  | 26 | 24.6 | 21.6 | Yes |  |
|  | Lorisidae |  | 10 | 35.4 | 32.4 | No |  |
|  | Megaladapidae |  | 3 | 27.3 | 15.3 | Yes |  |
|  | Palaeopropithecidae |  | 6 | 22.5 | 16.9 | Yes |  |
|  | Pitheciidae | Callicebinae | 32 | 20.2 | 18.0 | No |  |
|  |  | Pitheciinae | 8 | 20.2 | 16.9 | No |  |
|  | Tarsiidae |  | 10 | 62.4 | 20.6 | No |  |
| Proboscidea | Elephantidae | Elephantinae | 12 | 18.6 | 14.8 | No |  |
|  | Gomphotheriidae | Cuvieroniinae | 2 | 23.2 | 7.3 | No |  |
|  | Mammutidae |  | 1 | 26.0 | NA | No |  |
|  | Stegodontidae |  | 3 | 18.6 | 10.1 | No |  |
| Rodentia | Abrocomidae |  | 10 | 28.2 | 20.5 | No |  |
|  | Anomaluridae | Anomalurinae | 4 | 31.8 | 17.6 | No |  |
|  |  | Zenkerellinae | 3 | 51.8 | 40.0 | No |  |
|  | Aplodontidae | Aplodontinae | 1 | 49.1 | NA | No |  |
|  | Bathyergidae | Bathyerginae | 14 | 40.0 | 25.7 | No |  |
|  |  | Heterocephalinae | 1 | 40.0 | NA | No |  |
|  | Calomyscidae |  | 8 | 31.4 | 19.2 | No |  |
|  | Capromyidae | Capromyinae | 16 | 12.6 | 10.2 | Yes |  |
|  |  | Heptaxodontinae | 3 | 12.0 | 6.7 | Yes |  |
|  |  | Hexolobodontinae | 2 | 11.4 | 5.8 | Yes |  |
|  |  | Isolobodontinae | 2 | 12.3 | 6.7 | Yes |  |
|  |  | Plagiodontinae | 3 | 10.84 | 6.8 | Yes |  |
|  | Castoridae | Castorinae | 2 | 36.9 | 19.4 | No |  |
|  |  | Castoroidinae | 1 | 36.9 | NA | No |  |
|  | Caviidae | Caviinae | 12 | 19.9 | 18.2 | No |  |
|  |  | Dolichotinae | 2 | 16.2 | 9.5 | No |  |
|  |  | Hydrochoerinae | 5 | 16.2 | 12.7 | No |  |
|  | Chinchillidae |  | 7 | 28.2 | 22.0 | No |  |
|  | Cricetidae | Arvicolinae | 155 | 20.1 | 17.9 | No |  |
|  |  | Cricetinae | 21 | 20.1 | 16.1 | No |  |
|  |  | Neotominae | 128 | 24.4 | 21.4 | No |  |
|  |  | Sigmodontinae | 395 | 21.2 | 19.0 | No |  |
|  |  | Tylomyinae | 10 | 20.8 | 16.5 | No |  |
|  | Ctenodactylidae |  | 5 | 43.1 | 28.6 | No |  |
|  | Ctenomyidae |  | 60 | 22.9 | 20.6 | No |  |
|  | Cuniculidae |  | 2 | 32.2 | 10.4 | No |  |
|  | Dasyproctidae |  | 13 | 30.5 | 19.1 | No |  |
|  | Diatomyidae |  | 1 | 43.1 | NA | No |  |
|  | Dinomyidae |  | 1 | 28.2 | NA | No |  |
|  | Dipodidae |  | 50 | 54.2 | 47.0 | No |  |
|  | Echimyidae |  | 90 | 15.8 | 14.6 | No |  |
|  | Erethizontidae | Chaetomyinae | 1 | 25.7 | NA | No |  |
|  |  | Erethizontinae | 17 | 25.7 | 12.6 | No |  |
|  | Geomyidae |  | 39 | 32.6 | 27.1 | No |  |
|  | Gliridae | Glirinae | 2 | 30.0 | 15.7 | No |  |
|  |  | Graphiurinae | 14 | 28.3 | 22.4 | No |  |
|  |  | Leithiinae | 13 | 28.2 | 22.3 | No |  |
|  | Heteromyidae | Dipodomyinae | 23 | 29.7 | 16.0 | No |  |
|  |  | Heteromyinae | 13 | 25.3 | 14.4 | No |  |
|  |  | Perognathinae | 26 | 25.3 | 19.7 | No |  |
|  | Hystricidae |  | 13 | 49.0 | 27.5 | No |  |
|  | Muridae | Deomyinae | 35 | 19.5 | 16.2 | No |  |
|  |  | Gerbillinae | 100 | 19.9 | 17.8 | No |  |
|  |  | Leimacomyinae | 1 | 16.3 | NA | No |  |
|  |  | Lophiomyinae | 1 | 21.2 | NA | No |  |
|  |  | Murinae | 583 | 23.1 | 21.6 | No |  |
|  | Myocastoridae |  | 1 | 15.8 | NA | No |  |
|  | Nesomyidae | Cricetomyinae | 3 | 15.6 | 8.0 | No |  |
|  |  | Delanymyinae | 1 | 7.5 | NA | No |  |
|  |  | Dendromurinae | 23 | 12.7 | 9.0 | No |  |
|  |  | Mystromyinae | 1 | 13.2 | NA | No |  |
|  |  | Nesomyinae | 27 | 25.8 | 19.7 | Yes |  |
|  |  | Petromyscinae | 4 | 7.5 | 3.9 | No |  |
|  |  | Saccostomurinae | 2 | 12.7 | 4.4 | No |  |
|  | Octodontidae |  | 13 | 22.9 | 19.8 | No |  |
|  | Pedetidae |  | 2 | 51.8 | 5.0 | No |  |
|  | Petromuridae |  | 1 | 26.12 | NA | No |  |
|  | Platacanthomyidae |  | 2 | 49.6 | 24.2 | No |  |
|  | Sciuridae | Callosciurinae | 64 | 34.4 | 28.1 | No |  |
|  |  | Ratufinae | 4 | 17.6 | 10.0 | No |  |
|  |  | Sciurillinae | 1 | 17.6 | NA | No |  |
|  |  | Sciurinae | 81 | 28.6 | 23.9 | No |  |
|  |  | Xerinae | 129 | 36.0 | 30.3 | No |  |
|  | Spalacidae | Myospalacinae | 6 | 29.63 | 13.9 | No |  |
|  |  | Rhizomyinae | 6 | 43.3 | 28.4 | No |  |
|  |  | Spalacinae | 9 | 28.5 | 14.6 | No |  |
|  | Thryonomyidae |  | 2 | 26.2 | 14.3 | No |  |
| Scandentia | Ptilocercidae |  | 1 | 55.9 | NA | No |  |
|  | Tupaiidae |  | 19 | 55.9 | 35.0 | No |  |
| Tubulidentata | Orycteropodidae |  | 1 | 78.6 | NA | No |  |
| ^a^ Not analyed due to lack of body size data | | | | | | |  |

| **Table S4: Ecological traits of the analysed subfamilies.** | | | | | | | | | | | |
| --- | --- | --- | --- | --- | --- | --- | --- | --- | --- | --- | --- |
| Family | Subfamily | Range | Number of species | Body size (log10 weight in g) | Annual temperature (in °C) | Annual precipitation (log10 precipitation in mm) | Temperature niche width (in °C) | Precipitation niche width  (log10 precipitation in mm) | Rate of size evolution (log10 transformed rate of change per million years) | Rate of temperature preference evolution (log10 transformed rate of change per million years) | Rate of precipitation preference evolution (log10 transformed rate of change per million years) |
| *Insertae sedis* | Bibymalagasia | 67 | 1 | 4.121 | 23.507 | 3.146 | 3.531 | 0.266 | -0.988 | 1.614 | -3.394 |
| Chrysochloridae |  | 243 | 21 | 1.631 | 18.567 | 2.700 | 1.878 | 0.140 | -1.627 | 2.863 | -1.468 |
| Tenrecidae | Geogalinae | 35 | 1 | 0.845 | 24.704 | 2.939 | 2.031 | 0.297 | -0.597 | 1.725 | -2.853 |
|  | Oryzorictinae | 91 | 26 | 1.165 | 22.536 | 3.163 | 2.929 | 0.174 | -0.534 | 2.481 | -1.435 |
|  | Potamogalinae | 468 | 3 | 2.219 | 23.074 | 3.222 | 1.545 | 0.109 | -1.869 | 3.345 | -8.686 |
|  | Tenrecinae | 91 | 5 | 2.371 | 22.889 | 3.127 | 2.704 | 0.218 | -1.779 | 1.256 | -2.180 |
| Ailuridae | Ailurinae | 127 | 1 | 3.690 | 6.316 | 2.934 | 12.916 | 0.328 | -0.842 | 3.818 | -1.314 |
| Canidae | Caninae | 14682 | 41 | 3.825 | 16.538 | 2.733 | 6.341 | 0.385 | -1.534 | 5.520 | -0.007 |
| Eupleridae |  | 91 | 10 | 3.253 | 23.370 | 3.106 | 2.777 | 0.176 | -1.946 | 2.971 | -0.249 |
| Felidae | Felinae | 14362 | 41 | 4.085 | 17.343 | 2.881 | 7.581 | 0.430 | -1.523 | 4.154 | -1.165 |
|  | Machairodontinae | 4397 | 4 | 5.341 | 12.158 | 2.861 | 8.826 | 0.294 | -2.033 | 3.477 | -1.645 |
| Herpestidae |  | 4053 | 34 | 3.125 | 24.043 | 3.009 | 2.791 | 0.270 | -1.500 | 3.576 | -0.885 |
| Hyaenidae |  | 5901 | 4 | 4.482 | 21.904 | 2.589 | 6.991 | 0.615 | -1.535 | 3.761 | -1.026 |
| Mephitidae |  | 3281 | 13 | 3.004 | 17.435 | 2.910 | 6.625 | 0.325 | -1.250 | 5.191 | -0.701 |
| Mustelidae | Galictinae | 5172 | 7 | 2.924 | 19.666 | 2.756 | 5.894 | 0.448 | -0.390 | 4.281 | -1.119 |
|  | Helictidinae | 699 | 4 | 3.154 | 23.558 | 3.307 | 3.482 | 0.163 | -1.770 | 4.026 | -2.505 |
|  | Lutrinae | 10316 | 13 | 4.000 | 18.051 | 3.111 | 5.376 | 0.395 | 0.674 | 3.885 | 0.262 |
|  | Martinae | 8713 | 10 | 3.345 | 9.761 | 2.950 | 7.397 | 0.291 | -2.026 | 3.505 | -1.183 |
|  | Melinae | 3733 | 4 | 3.943 | 10.631 | 2.959 | 8.105 | 0.241 | -1.914 | 4.745 | -0.060 |
|  | Mellivorinae | 3480 | 1 | 3.929 | 24.953 | 2.787 | 4.856 | 0.889 | -1.298 | 3.704 | -1.082 |
|  | Mustelinae | 8907 | 19 | 2.617 | 11.240 | 2.903 | 6.745 | 0.231 | -1.583 | 5.730 | 0.966 |
|  | Taxidiinae | 1094 | 1 | 3.852 | 8.686 | 2.682 | 11.911 | 0.325 | -1.015 | 3.885 | -0.961 |
| Nandiniidae |  | 781 | 1 | 3.301 | 24.531 | 3.176 | 2.289 | 0.171 | -1.180 | 2.710 | -1.805 |
| Prionodontidae |  | 609 | 2 | 2.777 | 22.628 | 3.297 | 4.677 | 0.157 | -1.495 | 3.774 | -1.318 |
| Procyonidae |  | 3194 | 14 | 3.306 | 23.018 | 3.214 | 4.543 | 0.232 | -1.835 | 3.210 | -1.980 |
| Ursidae | Ailuropodinae | 211 | 1 | 5.035 | 15.711 | 3.054 | 4.534 | 0.190 | -0.888 | 4.228 | -0.988 |
|  | Tremarctinae | 3451 | 5 | 5.218 | 17.368 | 2.902 | 7.157 | 0.358 | -0.984 | 3.938 | 0.452 |
|  | Ursinae | 10470 | 7 | 5.138 | 9.915 | 2.861 | 8.662 | 0.318 | -1.526 | 3.898 | -1.723 |
| Viverridae | Hemigalinae | 314 | 5 | 3.449 | 25.303 | 3.382 | 2.019 | 0.124 | -2.723 | 2.833 | -1.610 |
|  | Paradoxurinae | 1419 | 6 | 3.561 | 25.239 | 3.239 | 3.956 | 0.309 | -2.604 | 2.115 | -3.272 |
|  | Viverrinae | 4027 | 22 | 3.451 | 24.509 | 3.149 | 3.007 | 0.230 | -2.200 | 2.912 | -1.411 |
| Antilocapridae | Antilocaprinae | 954 | 4 | 4.537 | 12.039 | 2.686 | 9.209 | 0.312 | -1.572 | 2.362 | -1.815 |
| Bovidae | Aepycerotini | 687 | 4 | 3.903 | 23.890 | 3.095 | 2.088 | 0.188 | -1.563 | 2.369 | -2.341 |
|  | Alcelaphini | 1762 | 10 | 5.118 | 20.882 | 2.828 | 3.158 | 0.269 | -1.706 | 4.561 | -1.635 |
|  | Antilopini | 5926 | 38 | 4.366 | 20.521 | 2.443 | 4.072 | 0.398 | -1.501 | 3.388 | -0.692 |
|  | Boselaphini | 331 | 2 | 4.753 | 25.805 | 3.011 | 1.519 | 0.265 | -1.153 | 2.710 | -1.931 |
|  | Bovini | 7495 | 15 | 5.696 | 19.500 | 3.077 | 4.665 | 0.234 | -0.575 | 3.113 | -1.846 |
|  | Caprini | 7327 | 43 | 4.799 | 10.592 | 2.757 | 6.670 | 0.300 | -0.810 | 4.445 | -0.494 |
|  | Cephalophini | 1912 | 18 | 4.267 | 24.679 | 3.157 | 1.891 | 0.183 | -2.017 | 3.020 | -1.605 |
|  | Hippotragini | 2513 | 8 | 5.189 | 23.187 | 2.439 | 4.138 | 0.350 | -0.459 | 4.148 | -1.234 |
|  | *Oreotragus* | 761 | 1 | 4.114 | 21.382 | 2.908 | 3.749 | 0.379 | -1.651 | 3.823 | -3.219 |
|  | Reduncini | 1510 | 9 | 4.804 | 22.847 | 2.960 | 2.914 | 0.206 | -1.826 | 4.674 | -2.846 |
|  | Tragelaphini | 1983 | 9 | 5.238 | 23.366 | 2.977 | 3.452 | 0.213 | 0.608 | 2.446 | 0.013 |
| Camelidae |  | 6375 | 10 | 5.553 | 14.619 | 2.719 | 7.105 | 0.396 | 0.603 | 4.559 | -1.821 |
| Cervidae | Capreolinae | 8837 | 33 | 4.621 | 15.735 | 3.051 | 5.799 | 0.261 | -1.805 | 3.477 | -1.607 |
|  | Cervinae | 6251 | 44 | 4.792 | 18.949 | 3.067 | 3.469 | 0.202 | -1.081 | 3.325 | -1.568 |
| Giraffidae | Giraffinae | 980 | 2 | 5.658 | 24.536 | 3.047 | 3.436 | 0.193 | -1.638 | 2.226 | -2.010 |
| Hippopotamidae |  | 2766 | 6 | 5.616 | 23.172 | 3.078 | 2.682 | 0.236 | -0.331 | 2.741 | -1.228 |
| Moschidae | Moschinae | 1350 | 7 | 4.086 | 5.150 | 2.834 | 8.489 | 0.408 | -3.626 | 3.051 | -1.062 |
| Suidae | Suinae | 6526 | 21 | 4.830 | 24.474 | 3.189 | 3.010 | 0.178 | -1.760 | 3.295 | -1.617 |
| Tayassuidae | Tayassuinae | 3387 | 6 | 4.661 | 18.890 | 3.047 | 6.383 | 0.284 | -1.774 | 3.120 | -1.027 |
| Tragulidae |  | 1051 | 10 | 3.445 | 25.892 | 3.264 | 1.588 | 0.152 | -2.786 | 2.185 | -1.770 |
| Craseonycteridae |  | 7 | 1 | 0.342 | 26.289 | 3.437 | 1.598 | 0.287 | -2.154 | 0.665 | -1.041 |
| Emballonuridae | Emballonurinae | 2933 | 34 | 0.828 | 25.439 | 3.336 | 2.192 | 0.189 | -1.472 | 2.366 | -2.367 |
|  | Taphozoinae | 3540 | 18 | 1.484 | 25.360 | 3.003 | 2.644 | 0.291 | -3.170 | 3.401 | -1.242 |
| Furipteridae |  | 1196 | 2 | 0.512 | 21.975 | 2.543 | 4.238 | 0.851 | -2.514 | 3.410 | -0.317 |
| Hipposideridae |  | 5561 | 83 | 1.108 | 24.855 | 3.241 | 2.312 | 0.163 | -2.193 | 2.492 | -0.527 |
| Megadermatidae |  | 2864 | 5 | 1.597 | 25.411 | 2.950 | 3.874 | 0.329 | -0.971 | -8.686 | -1.145 |
| Molossidae |  | 7779 | 100 | 1.287 | 23.761 | 3.128 | 3.020 | 0.244 | -2.568 | 2.227 | -1.505 |
| Mormoopidae |  | 1491 | 10 | 1.039 | 25.334 | 3.115 | 2.071 | 0.214 | -2.161 | 1.472 | -1.801 |
| Mystacinidae |  | 63 | 2 | 1.158 | 11.837 | 3.201 | 3.928 | 0.218 | -2.040 | 1.757 | -8.686 |
| Myzopodidae |  | 43 | 2 | 0.964 | 24.145 | 3.235 | 1.609 | 0.151 | -2.627 | 4.174 | -2.248 |
| Natalidae |  | 998 | 11 | 0.712 | 24.895 | 3.121 | 2.257 | 0.208 | -2.991 | 2.278 | -1.353 |
| Noctilionidae |  | 1835 | 2 | 1.627 | 25.401 | 3.248 | 2.623 | 0.251 | -1.773 | 0.919 | -8.686 |
| Nycteridae |  | 2586 | 16 | 1.072 | 24.766 | 3.083 | 2.531 | 0.181 | -1.893 | 2.638 | -1.526 |
| Phyllostomidae |  | 3452 | 178 | 1.263 | 24.364 | 3.242 | 3.195 | 0.231 | -1.750 | 3.597 | -0.928 |
| Pteropodidae |  | 4354 | 186 | 2.104 | 25.243 | 3.333 | 1.648 | 0.127 | -1.679 | 3.746 | -1.042 |
| Rhinolophidae |  | 5533 | 74 | 1.022 | 22.717 | 3.150 | 3.165 | 0.198 | -1.854 | 4.110 | -0.811 |
| Rhinopomatidae |  | 2388 | 4 | 1.123 | 25.524 | 2.275 | 4.370 | 0.668 | -2.288 | 2.508 | -1.620 |
| Thyropteridae |  | 1232 | 4 | 0.645 | 25.793 | 3.300 | 1.203 | 0.151 | -2.821 | -0.154 | -1.999 |
| Vespertilionidae | Kerivoulinae | 2168 | 25 | 0.761 | 24.973 | 3.285 | 1.954 | 0.138 | -0.621 | 3.323 | -1.237 |
|  | Miniopterinae | 4470 | 22 | 0.927 | 23.577 | 3.240 | 2.777 | 0.212 | -1.775 | 3.657 | -1.259 |
|  | Murininae | 1455 | 20 | 0.826 | 18.964 | 3.224 | 4.630 | 0.182 | -2.750 | 3.956 | -1.324 |
|  | Myotinae | 10057 | 102 | 0.867 | 17.381 | 2.996 | 4.640 | 0.219 | -2.034 | 3.937 | -0.161 |
|  | Vespertilioninae | 13719 | 242 | 0.943 | 20.647 | 3.019 | 3.826 | 0.227 | -2.181 | 3.689 | -0.272 |
| Dasypodidae | Chlamyphorinae | 110 | 2 | 1.880 | 19.547 | 2.705 | 1.894 | 0.197 | -1.708 | 2.976 | -2.584 |
| Dasypodidae | Dasypodinae | 2832 | 10 | 3.743 | 20.879 | 3.098 | 4.254 | 0.220 | -1.060 | 4.800 | -0.304 |
| Dasypodidae | Euphractinae | 1215 | 6 | 3.664 | 15.641 | 2.724 | 6.550 | 0.451 | -1.460 | 4.262 | -0.183 |
| Dasypodidae | Tolypeutinae | 1614 | 7 | 3.582 | 24.082 | 3.114 | 2.824 | 0.199 | -1.842 | 1.894 | -1.889 |
| Glyptodontidae |  | 2402 | 9 | 5.872 | 20.516 | 3.053 | 5.117 | 0.262 | -1.288 | 4.361 | -0.529 |
| Pampatheridae |  | 2161 | 5 | 5.255 | 21.994 | 3.076 | 4.671 | 0.313 | -1.814 | 4.345 | -0.411 |
| Dasyuridae | Dasyurinae | 1078 | 43 | 2.038 | 22.004 | 3.015 | 2.871 | 0.161 | -1.578 | 4.600 | -1.424 |
|  | Sminthopsinae | 957 | 29 | 1.183 | 21.400 | 2.669 | 2.171 | 0.173 | -2.444 | 2.976 | -0.759 |
| Myrmecobiidae |  | 240 | 1 | 2.673 | 18.917 | 2.377 | 3.129 | 0.222 | -1.538 | 3.128 | -1.175 |
| Thylacinidae |  | 999 | 1 | 4.477 | 22.055 | 2.612 | 6.615 | 0.507 | -1.337 | 3.181 | -1.004 |
| Cynocephalidae |  | 318 | 2 | 3.051 | 26.348 | 3.413 | 1.580 | 0.162 | -2.099 | 0.793 | -4.069 |
| Didelphidae | Caluromyinae | 1246 | 5 | 2.360 | 25.134 | 3.290 | 2.362 | 0.209 | -3.154 | -8.686 | -8.686 |
|  | Didelphinae | 2859 | 94 | 1.775 | 22.688 | 3.152 | 3.312 | 0.185 | -2.004 | 2.947 | -1.637 |
| Acrobatidae |  | 299 | 2 | 1.362 | 22.215 | 3.164 | 4.753 | 0.199 | -1.967 | 2.871 | -1.176 |
| Burramyidae |  | 281 | 5 | 1.271 | 15.705 | 2.973 | 2.901 | 0.215 | -0.652 | 2.632 | -1.663 |
| Diprotodontidae | Diprotodontinae | 292 | 1 | 6.431 | 17.432 | 2.752 | 3.540 | 0.358 | -1.318 | 2.904 | -1.178 |
|  | Palorchestinae | 67 | 1 | 5.699 | 13.360 | 2.952 | 2.581 | 0.162 | -1.538 | 2.970 | -1.674 |
|  | Zygomaturinae | 133 | 2 | 5.349 | 19.073 | 3.165 | 3.728 | 0.211 | -0.202 | 3.959 | -0.221 |
| Hypsiprymnodontidae |  | 176 | 2 | 3.659 | 20.408 | 2.985 | 2.803 | 0.220 | -0.279 | 3.437 | -0.860 |
| Macropodidae | Lagostrophinae | 209 | 2 | 3.916 | 16.579 | 2.720 | 4.950 | 0.217 | -0.033 | 2.245 | -1.205 |
|  | Macropodinae | 1106 | 75 | 3.912 | 22.139 | 3.050 | 2.938 | 0.174 | -1.662 | 3.741 | -1.359 |
|  | Sthenurinae | 345 | 9 | 4.998 | 16.234 | 2.790 | 3.465 | 0.253 | -0.178 | 3.553 | -1.237 |
| Petauridae |  | 482 | 12 | 2.400 | 22.659 | 3.281 | 3.232 | 0.162 | -1.682 | 2.426 | -1.099 |
| Phalangeridae |  | 1180 | 26 | 3.389 | 24.782 | 3.347 | 1.808 | 0.114 | -1.751 | 2.342 | -2.227 |
| Phascolarctidae |  | 236 | 1 | 4.011 | 19.262 | 2.810 | 6.089 | 0.261 | -1.135 | 2.685 | -1.770 |
| Potoroidae |  | 732 | 12 | 3.206 | 17.684 | 2.703 | 2.953 | 0.190 | -2.015 | 3.049 | 0.088 |
| Pseudocheiridae |  | 403 | 19 | 2.902 | 22.941 | 3.299 | 3.357 | 0.165 | -1.984 | 2.050 | -2.018 |
| Tarsipedidae |  | 50 | 1 | 0.954 | 16.901 | 2.617 | 2.091 | 0.225 | -1.829 | 2.033 | -2.345 |
| Thylacoleonidae |  | 285 | 1 | 5.041 | 17.159 | 2.803 | 4.036 | 0.380 | -1.117 | 2.705 | -1.464 |
| Vombatidae |  | 347 | 6 | 4.633 | 17.004 | 2.712 | 3.117 | 0.250 | -1.420 | 1.330 | -0.909 |
| Erinaceidae | Erinaceinae | 5025 | 16 | 2.570 | 16.734 | 2.581 | 5.160 | 0.337 | -2.164 | 3.674 | -0.254 |
|  | Galericinae | 453 | 8 | 2.039 | 24.001 | 3.349 | 2.163 | 0.132 | -1.534 | 2.263 | -2.036 |
| Nesophontidae |  | 59 | 8 | 1.544 | 25.433 | 3.141 | 0.986 | 0.090 | -1.078 | 0.721 | -8.686 |
| Solenodontidae |  | 50 | 3 | 2.710 | 25.057 | 3.141 | 1.421 | 0.125 | -2.143 | 0.246 | -8.686 |
| Soricidae | Crocidurinae | 6847 | 211 | 1.001 | 22.199 | 3.037 | 2.478 | 0.176 | -0.561 | 4.274 | 0.080 |
|  | Myosoricinae | 159 | 19 | 1.045 | 20.203 | 3.076 | 1.349 | 0.089 | -3.488 | 3.565 | -0.309 |
|  | Soricinae | 6542 | 148 | 0.894 | 12.187 | 2.973 | 4.982 | 0.181 | -2.234 | 4.281 | -0.829 |
| Talpidae |  | 3227 | 41 | 1.772 | 12.166 | 3.026 | 4.526 | 0.168 | -1.811 | 4.205 | -1.091 |
| Procaviidae |  | 2582 | 5 | 3.452 | 23.483 | 2.953 | 3.343 | 0.365 | -8.686 | 3.242 | -1.810 |
| Leporidae |  | 12357 | 63 | 3.262 | 15.614 | 2.825 | 4.362 | 0.221 | -1.950 | 4.071 | -0.580 |
| Ochotonidae |  | 3345 | 31 | 2.213 | 1.935 | 2.593 | 6.001 | 0.313 | -1.900 | 4.202 | -0.614 |
| Prolagidae |  | 10 | 1 | 2.544 | 14.585 | 2.814 | 1.971 | 0.056 | -2.330 | 2.992 | -1.676 |
| Macraucheniidae | Macraucheniinae | 1608 | 2 | 5.937 | 22.945 | 3.134 | 4.003 | 0.288 | -2.044 | 2.686 | -8.686 |
| Proterotheriidae | Megadolodinae | 1359 | 1 | 4.673 | 25.349 | 3.255 | 3.052 | 0.236 | -2.044 | 2.511 | -8.686 |
| Macroscelididae |  | 1289 | 19 | 2.015 | 21.272 | 2.649 | 2.639 | 0.234 | -1.719 | 2.008 | -1.577 |
| Microbiotheriidae |  | 29 | 1 | 1.348 | 10.067 | 3.148 | 2.702 | 0.254 | -1.648 | 2.220 | -1.540 |
| Ornithorhynchidae |  | 158 | 1 | 3.172 | 16.790 | 2.923 | 7.568 | 0.205 | -0.997 | 2.563 | -1.311 |
| Tachyglossidae |  | 1064 | 6 | 3.920 | 22.087 | 3.072 | 3.126 | 0.197 | 0.186 | 2.444 | -1.553 |
| Notoryctidae |  | 195 | 2 | 1.636 | 23.645 | 2.420 | 2.165 | 0.220 | -2.218 | 3.415 | -2.126 |
| Toxodontidae | Toxodontinae | 1928 | 3 | 6.108 | 24.338 | 3.158 | 5.007 | 0.331 | -2.044 | 1.829 | -8.686 |
| Caenolestidae |  | 88 | 6 | 1.496 | 17.749 | 3.288 | 5.244 | 0.187 | -3.055 | 2.097 | -1.704 |
| Chaeropodidae |  | 375 | 1 | 2.301 | 21.357 | 2.374 | 4.501 | 0.190 | -0.990 | 2.186 | -2.224 |
| Peramelidae | Echymiperinae | 192 | 10 | 2.673 | 24.806 | 3.451 | 2.228 | 0.107 | -1.848 | 1.168 | -1.715 |
| Peramelidae | Peramelinae | 869 | 7 | 2.758 | 19.761 | 2.728 | 4.493 | 0.238 | -1.681 | 3.812 | -1.125 |
| Peramelidae | Peroryctinae | 83 | 4 | 3.193 | 25.503 | 3.392 | 1.790 | 0.092 | -0.512 | 2.745 | -8.686 |
| Thylacomyidae |  | 663 | 2 | 2.840 | 22.226 | 2.417 | 3.777 | 0.247 | -0.362 | 1.894 | -2.250 |
| Equidae |  | 11522 | 12 | 5.475 | 12.910 | 2.598 | 7.429 | 0.400 | -1.376 | 5.381 | -0.988 |
| Rhinocerotidae | Elasmotheriinae | 446 | 1 | 6.613 | 2.769 | 2.511 | 4.309 | 0.236 | -1.506 | 3.144 | -2.313 |
|  | Rhinocerotinae | 5197 | 8 | 6.319 | 16.860 | 2.937 | 6.655 | 0.270 | -2.266 | 2.780 | -2.080 |
| Tapiridae |  | 3558 | 8 | 5.432 | 20.677 | 3.147 | 5.436 | 0.259 | -2.981 | 3.017 | -1.839 |
| Manidae |  | 2758 | 9 | 3.864 | 24.628 | 3.202 | 2.455 | 0.183 | -0.227 | 3.207 | -0.594 |
| Bradypodidae |  | 1329 | 4 | 3.573 | 24.754 | 3.297 | 1.258 | 0.111 | -1.797 | 2.263 | -2.804 |
| Cyclopedidae |  | 994 | 1 | 2.518 | 25.845 | 3.345 | 1.424 | 0.156 | -1.058 | 2.866 | -1.776 |
| Megalonychidae | Megalonychinae | 2494 | 13 | 4.507 | 22.620 | 3.116 | 2.871 | 0.194 | -1.584 | 2.897 | -1.930 |
| Megatheriidae | Megatheriinae | 2469 | 3 | 6.538 | 22.443 | 3.136 | 6.024 | 0.270 | -1.422 | 3.998 | -1.086 |
| Mylodontidae | Lestodontinae | 1025 | 2 | 6.382 | 22.289 | 3.087 | 5.097 | 0.312 | -0.979 | 3.907 | -0.967 |
|  | Mylodontinae | 1528 | 2 | 6.172 | 9.382 | 2.575 | 9.304 | 0.441 | -1.158 | 4.209 | -0.453 |
|  | Scelidotheriinae | 1672 | 4 | 5.698 | 20.004 | 2.983 | 4.808 | 0.360 | 0.943 | 5.178 | 0.330 |
| Myrmecophagidae |  | 1778 | 3 | 3.905 | 25.234 | 3.236 | 2.914 | 0.267 | -1.486 | 1.494 | -8.686 |
| Nothrotheridae |  | 1584 | 2 | 5.327 | 18.546 | 2.914 | 6.106 | 0.225 | -1.422 | 3.998 | -1.086 |
| Aotidea |  | 955 | 11 | 2.948 | 24.515 | 3.335 | 3.319 | 0.186 | -8.686 | 3.500 | -0.570 |
| Archaeolemuridae |  | 67 | 3 | 4.408 | 22.700 | 3.111 | 2.912 | 0.291 | -1.973 | 2.953 | -1.859 |
| Atelidae | Alouattinae | 1517 | 12 | 3.813 | 25.420 | 3.246 | 1.686 | 0.145 | -8.686 | 1.816 | -1.099 |
|  | Atelinae | 1559 | 16 | 3.902 | 24.659 | 3.296 | 2.641 | 0.154 | -2.130 | 1.709 | -2.347 |
| Callitrichidae |  | 1101 | 41 | 2.607 | 25.250 | 3.298 | 1.039 | 0.100 | -2.666 | 1.272 | -2.982 |
| Cebidae | Cebinae | 1281 | 9 | 3.458 | 24.275 | 3.174 | 1.777 | 0.135 | -8.686 | 2.452 | -1.420 |
|  | Saimiriinae | 803 | 5 | 2.798 | 25.710 | 3.395 | 1.913 | 0.113 | -0.332 | 2.571 | -1.129 |
| Cercopithecidae | Cercopithecinae | 4253 | 67 | 3.794 | 23.929 | 3.175 | 2.595 | 0.194 | -2.363 | 3.763 | -1.829 |
|  | Colobinae | 1940 | 56 | 3.942 | 23.443 | 3.269 | 2.955 | 0.144 | -2.864 | 3.073 | -2.055 |
| Cheirogaleidae |  | 90 | 32 | 2.045 | 23.386 | 3.184 | 1.759 | 0.112 | -2.425 | 2.612 | -2.522 |
| Daubentoniidae |  | 85 | 2 | 3.772 | 23.146 | 3.158 | 3.251 | 0.303 | -1.580 | 1.927 | -8.686 |
| Galagidae |  | 1947 | 18 | 2.306 | 24.519 | 3.071 | 2.299 | 0.165 | -2.152 | 3.727 | -2.028 |
| Hominidae |  | 9532 | 10 | 4.782 | 20.721 | 3.080 | 5.522 | 0.283 | -2.112 | 4.279 | -1.945 |
| Hylobatidae |  | 507 | 16 | 3.832 | 24.587 | 3.326 | 2.135 | 0.121 | -3.792 | 2.326 | -2.392 |
| Indriidae |  | 90 | 19 | 3.362 | 23.493 | 3.226 | 2.867 | 0.155 | -2.483 | 2.258 | -2.478 |
| Lemuridae |  | 91 | 23 | 3.341 | 23.173 | 3.180 | 2.553 | 0.175 | -2.583 | 3.865 | -1.749 |
| Lepilemuridae |  | 89 | 26 | 2.915 | 24.220 | 3.126 | 1.965 | 0.124 | -8.686 | 3.955 | -2.304 |
| Lorisidae |  | 1105 | 10 | 2.731 | 25.584 | 3.298 | 2.160 | 0.170 | -2.180 | 1.759 | -1.737 |
| Megaladapidae |  | 81 | 3 | 4.822 | 23.125 | 3.095 | 2.973 | 0.246 | -1.862 | 2.370 | -2.173 |
| Palaeopropithecidae |  | 81 | 6 | 4.500 | 22.231 | 3.178 | 2.874 | 0.232 | -1.418 | 2.677 | -1.383 |
| Pitheciidae | Callicebinae | 825 | 32 | 3.059 | 25.224 | 3.278 | 1.565 | 0.098 | -2.846 | 1.044 | -2.760 |
|  | Pitheciinae | 513 | 8 | 3.420 | 26.108 | 3.377 | 1.029 | 0.085 | -3.011 | -8.686 | -3.752 |
| Tarsiidae |  | 229 | 10 | 2.038 | 25.224 | 3.370 | 1.366 | 0.087 | -1.566 | 2.220 | -1.122 |
| Elephantidae | Elephantinae | 10111 | 12 | 6.270 | 15.377 | 2.755 | 4.893 | 0.272 | 0.731 | 3.208 | -0.420 |
| Gomphotheriidae | Cuvieroniinae | 1790 | 2 | 6.789 | 20.026 | 2.914 | 6.026 | 0.365 | -1.511 | 3.992 | -0.401 |
| Mammutidae |  | 1646 | 1 | 6.656 | 7.956 | 2.865 | 13.817 | 0.381 | -0.327 | 3.452 | -1.310 |
| Stegodontidae |  | 429 | 3 | 6.122 | 24.322 | 3.222 | 3.712 | 0.160 | -1.340 | 2.497 | -2.515 |
| Abrocomidae |  | 104 | 10 | 2.465 | 12.003 | 2.585 | 3.156 | 0.178 | -3.056 | 4.582 | -1.776 |
| Anomaluridae | Anomalurinae | 748 | 4 | 2.762 | 24.838 | 3.202 | 1.568 | 0.111 | -1.269 | 2.060 | -8.686 |
|  | Zenkerellinae | 158 | 3 | 1.926 | 24.210 | 3.234 | 1.514 | 0.082 | -1.653 | 2.018 | -8.686 |
| Aplodontidae | Aplodontinae | 60 | 1 | 3.002 | 8.945 | 3.059 | 3.629 | 0.475 | -1.230 | 3.165 | -1.736 |
| Bathyergidae | Bathyerginae | 886 | 14 | 2.219 | 20.943 | 2.858 | 1.955 | 0.152 | -1.853 | 1.822 | -1.554 |
|  | Heterocephalinae | 185 | 1 | 1.740 | 26.346 | 2.553 | 3.378 | 0.365 | -1.456 | 2.051 | -1.487 |
| Calomyscidae |  | 187 | 8 | 1.234 | 14.683 | 2.388 | 4.467 | 0.216 | -8.686 | 3.178 | -1.241 |
| Capromyidae | Capromyinae | 46 | 16 | 3.074 | 25.618 | 3.108 | 0.480 | 0.049 | -1.922 | 0.319 | -1.743 |
|  | Heptaxodontinae | 26 | 3 | 3.598 | 25.707 | 3.132 | 1.242 | 0.109 | 0.453 | 2.419 | -8.686 |
|  | Hexolobodontinae | 20 | 2 | 3.500 | 24.984 | 3.152 | 1.723 | 0.146 | -1.020 | 0.670 | -8.686 |
|  | Isolobodontinae | 20 | 2 | 3.099 | 24.648 | 3.152 | 1.686 | 0.131 | -1.337 | 1.047 | -8.686 |
|  | Plagiodontinae | 20 | 3 | 3.447 | 24.984 | 3.152 | 1.723 | 0.146 | -1.629 | 0.242 | -8.686 |
| Castoridae | Castorinae | 4625 | 2 | 4.309 | 4.297 | 2.734 | 11.496 | 0.327 | -1.753 | 1.964 | -1.895 |
|  | Castoroidinae | 1943 | 1 | 5.176 | 2.091 | 2.742 | 12.220 | 0.388 | -1.555 | 2.120 | -1.543 |
| Caviidae | Caviinae | 1322 | 12 | 2.599 | 16.829 | 2.877 | 4.439 | 0.286 | -1.961 | 3.883 | -2.031 |
|  | Dolichotinae | 221 | 2 | 3.554 | 17.775 | 2.603 | 4.929 | 0.245 | -1.123 | 2.399 | -2.272 |
|  | Hydrochoerinae | 2186 | 5 | 4.066 | 24.958 | 3.165 | 2.939 | 0.233 | -1.371 | -8.686 | -2.854 |
| Chinchillidae |  | 430 | 7 | 3.206 | 9.793 | 2.606 | 7.791 | 0.642 | -2.175 | 3.023 | -2.197 |
| Cricetidae | Arvicolinae | 7622 | 155 | 1.612 | 5.609 | 2.758 | 5.027 | 0.201 | -2.139 | 3.843 | -1.006 |
|  | Cricetinae | 2435 | 21 | 1.798 | 5.141 | 2.548 | 4.411 | 0.283 | -1.810 | 2.724 | -1.811 |
|  | Neotominae | 1972 | 128 | 1.626 | 19.205 | 2.877 | 3.694 | 0.172 | -2.229 | 3.790 | -1.212 |
|  | Sigmodontinae | 2800 | 395 | 1.700 | 19.584 | 3.050 | 3.894 | 0.225 | -1.824 | 3.157 | -1.012 |
|  | Tylomyinae | 190 | 10 | 2.160 | 24.985 | 3.302 | 1.245 | 0.147 | -2.334 | 2.371 | -1.064 |
| Ctenodactylidae |  | 266 | 5 | 2.302 | 23.556 | 2.011 | 2.526 | 0.388 | -8.686 | 2.134 | -1.176 |
| Ctenomyidae |  | 387 | 60 | 2.315 | 15.609 | 2.774 | 1.993 | 0.168 | -1.684 | 3.473 | -1.340 |
| Cuniculidae |  | 1538 | 2 | 3.929 | 23.412 | 3.266 | 4.810 | 0.231 | -2.094 | 2.960 | -2.569 |
| Dasyproctidae |  | 1399 | 13 | 3.350 | 25.358 | 3.306 | 2.181 | 0.148 | -2.648 | 3.383 | -0.866 |
| Diatomyidae |  | 3 | 1 | 2.574 | 24.099 | 3.378 | 1.903 | 0.062 | -3.616 | 2.350 | -1.766 |
| Dinomyidae |  | 282 | 1 | 4.097 | 25.578 | 3.373 | 2.160 | 0.159 | -2.021 | 2.230 | -2.169 |
| Dipodidae |  | 5133 | 50 | 1.477 | 8.297 | 2.412 | 4.430 | 0.249 | -2.400 | 3.185 | -1.632 |
| Echimyidae |  | 1612 | 90 | 2.413 | 23.938 | 3.219 | 1.620 | 0.120 | -1.743 | 3.027 | -1.539 |
| Erethizontidae | Chaetomyinae | 32 | 1 | 3.114 | 23.603 | 3.076 | 1.093 | 0.104 | -1.522 | 3.091 | -1.980 |
|  | Erethizontinae | 3103 | 17 | 3.232 | 22.501 | 3.240 | 3.925 | 0.172 | -1.925 | 2.629 | -2.349 |
| Geomyidae |  | 1040 | 39 | 2.364 | 17.407 | 2.980 | 2.980 | 0.141 | -2.041 | 2.632 | -1.485 |
| Gliridae | Glirinae | 645 | 2 | 1.764 | 11.474 | 3.037 | 4.339 | 0.142 | -0.741 | 2.345 | -1.371 |
|  | Graphiurinae | 1383 | 14 | 1.434 | 22.245 | 2.985 | 2.233 | 0.208 | -1.994 | 2.825 | -1.025 |
|  | Leithiinae | 1556 | 13 | 1.582 | 12.077 | 2.606 | 3.957 | 0.196 | -1.684 | 2.781 | -1.224 |
| Heteromyidae | Dipodomyinae | 592 | 23 | 1.747 | 15.519 | 2.529 | 3.172 | 0.220 | -2.389 | 2.537 | -0.982 |
|  | Heteromyinae | 379 | 13 | 1.770 | 23.653 | 3.161 | 4.123 | 0.195 | -3.100 | 2.462 | -1.088 |
|  | Perognathinae | 737 | 26 | 1.216 | 17.015 | 2.523 | 4.442 | 0.269 | -2.947 | 3.222 | -1.826 |
| Hystricidae |  | 3723 | 13 | 3.811 | 23.275 | 3.156 | 4.253 | 0.253 | -1.777 | 3.795 | -1.768 |
| Muridae | Deomyinae | 2519 | 35 | 1.594 | 22.793 | 2.800 | 2.463 | 0.222 | -2.148 | 3.262 | -1.135 |
|  | Gerbillinae | 5413 | 100 | 1.608 | 22.053 | 2.320 | 3.063 | 0.358 | -1.581 | 3.573 | 0.311 |
|  | Leimacomyinae | 1 | 1 | 1.778 | 26.378 | 3.114 | 0.000 | 0.000 | -1.381 | 3.238 | -1.538 |
|  | Lophiomyinae | 80 | 1 | 2.878 | 23.245 | 2.711 | 5.983 | 0.599 | -1.847 | 3.022 | -1.716 |
|  | Murinae | 9022 | 583 | 1.897 | 22.799 | 3.177 | 2.503 | 0.142 | -1.654 | 3.223 | -1.593 |
| Myocastoridae |  | 512 | 1 | 3.841 | 16.454 | 2.898 | 8.319 | 0.555 | -1.679 | 1.954 | -2.694 |
| Nesomyidae | Cricetomyinae | 1447 | 3 | 2.699 | 24.261 | 3.086 | 3.110 | 0.148 | -1.335 | 1.688 | -8.686 |
|  | Delanymyinae | 9 | 1 | 0.699 | 19.674 | 3.120 | 1.131 | 0.135 | -1.695 | 3.443 | -1.741 |
|  | Dendromurinae | 1233 | 23 | 1.227 | 22.013 | 3.034 | 2.118 | 0.152 | -1.335 | 3.732 | -0.163 |
|  | Mystromyinae | 86 | 1 | 1.939 | 16.377 | 2.768 | 2.268 | 0.229 | -1.471 | 3.390 | -1.378 |
|  | Nesomyinae | 91 | 27 | 1.987 | 22.565 | 3.164 | 2.334 | 0.159 | -1.810 | 3.515 | -0.714 |
|  | Petromyscinae | 108 | 4 | 1.397 | 18.936 | 2.334 | 1.899 | 0.231 | -1.695 | 3.443 | -1.741 |
|  | Saccostomurinae | 764 | 2 | 1.684 | 22.916 | 2.811 | 4.424 | 0.332 | -2.065 | 3.562 | -2.621 |
| Octodontidae |  | 140 | 13 | 2.149 | 12.978 | 2.690 | 4.476 | 0.339 | -2.584 | 2.478 | -1.440 |
| Pedetidae |  | 562 | 2 | 3.522 | 21.175 | 2.870 | 2.396 | 0.255 | -2.038 | 0.933 | -3.436 |
| Petromuridae |  | 69 | 1 | 2.350 | 19.647 | 2.254 | 2.345 | 0.369 | -1.734 | 2.292 | -1.898 |
| Platacanthomyidae |  | 175 | 2 | 1.632 | 21.526 | 3.231 | 3.135 | 0.295 | -1.022 | 3.424 | -1.820 |
| Sciuridae | Callosciurinae | 1482 | 64 | 2.193 | 24.473 | 3.343 | 2.635 | 0.144 | -2.168 | 4.388 | -0.622 |
|  | Ratufinae | 639 | 4 | 3.131 | 26.089 | 3.264 | 2.269 | 0.201 | -1.704 | 2.752 | -1.934 |
|  | Sciurillinae | 154 | 1 | 1.591 | 26.222 | 3.370 | 0.822 | 0.074 | -1.704 | 2.752 | -1.934 |
|  | Sciurinae | 7493 | 81 | 2.487 | 19.991 | 3.193 | 4.487 | 0.204 | -0.635 | 4.660 | 0.296 |
|  | Xerinae | 8020 | 129 | 2.471 | 12.632 | 2.764 | 3.965 | 0.228 | -1.773 | 3.452 | -1.232 |
| Spalacidae | Myospalacinae | 585 | 6 | 2.446 | 5.685 | 2.715 | 5.164 | 0.194 | -8.686 | 2.269 | -2.752 |
|  | Rhizomyinae | 731 | 6 | 2.836 | 21.193 | 3.148 | 4.696 | 0.170 | -1.436 | 3.739 | -2.455 |
|  | Spalacinae | 449 | 9 | 2.485 | 10.212 | 2.632 | 2.555 | 0.174 | -2.401 | 2.094 | -0.634 |
| Thryonomyidae |  | 986 | 2 | 3.467 | 22.857 | 3.024 | 3.245 | 0.212 | -2.082 | 2.172 | -2.038 |
| Ptilocercidae |  | 96 | 1 | 1.622 | 26.508 | 3.442 | 1.533 | 0.130 | -2.627 | 1.876 | -8.686 |
| Tupaiidae |  | 897 | 19 | 2.174 | 26.083 | 3.402 | 1.697 | 0.116 | -2.620 | 1.719 | -0.582 |
| Orycteropodidae |  | 2236 | 1 | 4.719 | 24.559 | 2.945 | 5.354 | 0.474 | -1.644 | 1.510 | -2.664 |

| **Table S5: Model residuals.** | | | | |
| --- | --- | --- | --- | --- |
| Order | Family | Subfamily | Standardised residuals from full model | Standardised residuals from models excluding island endemic clades |
| Afrosoricida | *Insertae sedis* | Bibymalagasia | -0.784 | NA |
|  | Chrysochloridae |  | -0.597 | -0.754 |
|  | Tenrecidae | Geogalinae | -0.490 | NA |
|  |  | Oryzorictinae | -1.375 | NA |
|  |  | Potamogalinae | 0.669 | 0.185 |
|  |  | Tenrecinae | -0.877 | NA |
| Carnivora | Ailuridae | Ailurinae | -1.078 | -1.156 |
|  | Canidae | Caninae | 0.288 | 0.222 |
|  | Eupleridae |  | -1.256 | NA |
|  | Felidae | Felinae | -0.043 | -0.204 |
|  |  | Machairodontinae | 0.353 | 0.281 |
|  | Herpestidae |  | 0.171 | 0.004 |
|  | Hyaenidae |  | 0.106 | 0.107 |
|  | Mephitidae |  | 0.200 | 0.120 |
|  | Mustelidae | Galictinae | 0.535 | 0.510 |
|  |  | Helictidinae | 0.382 | 0.168 |
|  |  | Lutrinae | 0.698 | 0.611 |
|  |  | Martinae | 0.944 | 0.900 |
|  |  | Melinae | 0.824 | 0.853 |
|  |  | Mellivorinae | 0.410 | 0.406 |
|  |  | Mustelinae | 0.999 | 1.050 |
|  |  | Taxidiinae | 0.141 | 0.231 |
|  | Nandiniidae |  | 1.129 | 1.083 |
|  | Prionodontidae |  | 0.601 | 0.519 |
|  | Procyonidae |  | 0.339 | 0.142 |
|  | Ursidae | Ailuropodinae | 0.013 | -0.108 |
|  |  | Tremarctinae | 0.139 | 0.118 |
|  |  | Ursinae | 0.666 | 0.577 |
|  | Viverridae | Hemigalinae | -0.051 | -0.279 |
|  |  | Paradoxurinae | 0.100 | -0.154 |
|  |  | Viverrinae | 0.359 | 0.177 |
| Cetartiodactyla | Antilocapridae | Antilocaprinae | -0.549 | -0.636 |
|  | Bovidae | Aepycerotini | 0.236 | 0.044 |
|  |  | Alcelaphini | -0.053 | -0.247 |
|  |  | Antilopini | -0.312 | -0.405 |
|  |  | Boselaphini | -0.106 | -0.292 |
|  |  | Bovini | 0.368 | 0.136 |
|  |  | Caprini | -0.123 | -0.286 |
|  |  | Cephalophini | 0.082 | -0.162 |
|  |  | Hippotragini | -0.192 | -0.266 |
|  |  | *Oreotragus* | 0.511 | 0.377 |
|  |  | Reduncini | 0.067 | -0.193 |
|  |  | Tragelaphini | -0.027 | -0.150 |
|  | Camelidae |  | 0.027 | -0.145 |
|  | Cervidae | Capreolinae | 0.222 | 0.001 |
|  |  | Cervinae | 0.126 | -0.145 |
|  | Giraffidae | Giraffinae | 0.253 | 0.105 |
|  | Hippopotamidae |  | 0.398 | 0.219 |
|  | Moschidae | Moschinae | -0.303 | -0.408 |
|  | Suidae | Suinae | 0.498 | 0.275 |
|  | Tayassuidae | Tayassuinae | 0.308 | 0.209 |
|  | Tragulidae |  | 0.240 | 0.032 |
| Chiroptera | Craseonycteridae |  | -1.154 | -1.330 |
|  | Emballonuridae | Emballonurinae | 0.594 | 0.369 |
|  |  | Taphozoinae | 0.678 | 0.613 |
|  | Furipteridae |  | 0.254 | 0.378 |
|  | Hipposideridae |  | 0.523 | 0.380 |
|  | Megadermatidae |  | 0.453 | 0.541 |
|  | Molossidae |  | 0.362 | 0.178 |
|  | Mormoopidae |  | 0.675 | 0.589 |
|  | Mystacinidae |  | -0.309 | NA |
|  | Myzopodidae |  | -0.238 | NA |
|  | Natalidae |  | 0.506 | 0.434 |
|  | Noctilionidae |  | 1.272 | 0.897 |
|  | Nycteridae |  | 0.796 | 0.718 |
|  | Phyllostomidae |  | -0.329 | -0.601 |
|  | Pteropodidae |  | -0.021 | -0.331 |
|  | Rhinolophidae |  | 0.518 | 0.372 |
|  | Rhinopomatidae |  | 0.228 | 0.343 |
|  | Thyropteridae |  | 1.280 | 1.244 |
|  | Vespertilionidae | Kerivoulinae | 0.726 | 0.577 |
|  |  | Miniopterinae | 1.020 | 0.915 |
|  |  | Murininae | 0.398 | 0.268 |
|  |  | Myotinae | 0.625 | 0.554 |
|  |  | Vespertilioninae | 0.361 | 0.227 |
| Cingulata | Dasypodidae | Chlamyphorinae | -0.056 | -0.149 |
|  |  | Dasypodinae | 0.505 | 0.405 |
|  |  | Euphractinae | -0.319 | -0.363 |
|  |  | Tolypeutinae | 0.396 | 0.242 |
|  | Glyptodontidae |  | -0.123 | -0.301 |
|  | Pampatheridae |  | 0.177 | 0.058 |
| Dasyuromorphia | Dasyuridae | Dasyurinae | -0.264 | NA |
|  |  | Sminthopsinae | -0.027 | NA |
|  | Myrmecobiidae |  | 0.349 | NA |
|  | Thylacinidae |  | -0.045 | NA |
| Dermoptera | Cynocephalidae |  | 0.343 | 0.061 |
| Didelphimorphia | Didelphidae | Caluromyinae | 0.229 | -0.189 |
|  |  | Didelphinae | -0.172 | -0.428 |
| Diprotodontia | Acrobatidae |  | 0.331 | NA |
|  | Burramyidae |  | 0.152 | NA |
|  | Diprotodontidae | Diprotodontinae | -0.364 | NA |
|  |  | Palorchestinae | -0.500 | NA |
|  |  | Zygomaturinae | -0.657 | NA |
|  | Hypsiprymnodontidae |  | -0.193 | NA |
|  | Macropodidae | Lagostrophinae | -0.426 | NA |
|  |  | Macropodinae | -0.927 | NA |
|  |  | Sthenurinae | -0.874 | NA |
|  | Petauridae |  | -0.231 | NA |
|  | Phalangeridae |  | -0.090 | NA |
|  | Phascolarctidae |  | -0.259 | NA |
|  | Potoroidae |  | -0.127 | NA |
|  | Pseudocheiridae |  | -0.700 | NA |
|  | Tarsipedidae |  | -0.044 | NA |
|  | Thylacoleonidae |  | -0.206 | NA |
|  | Vombatidae |  | -0.657 | NA |
| Eulipotyphla | Erinaceidae | Erinaceinae | 0.479 | 0.521 |
|  |  | Galericinae | 0.141 | -0.081 |
|  | Nesophontidae |  | -0.986 | NA |
|  | Solenodontidae |  | -0.911 | NA |
|  | Soricidae | Crocidurinae | 0.226 | 0.066 |
|  |  | Myosoricinae | -0.411 | -0.552 |
|  |  | Soricinae | 0.342 | 0.190 |
|  | Talpidae |  | 0.486 | 0.337 |
| Hyracoidea | Procaviidae |  | 0.675 | 0.648 |
| Lagomorpha | Leporidae |  | 0.567 | 0.462 |
|  | Ochotonidae |  | 0.328 | 0.268 |
|  | Prolagidae |  | -0.831 | -0.958 |
| Litopterna | Macraucheniidae | Macraucheniinae | 0.272 | -0.255 |
|  | Proterotheriidae | Megadolodinae | 0.912 | 0.460 |
| Macroscelidea | Macroscelididae |  | -0.012 | -0.094 |
| Microbiotheria | Microbiotheriidae |  | -0.207 | -0.348 |
| Monotrremata | Ornithorhynchidae |  | -0.337 | NA |
|  | Tachyglossidae |  | 0.171 | NA |
| Notoryctemorphia | Notoryctidae |  | 0.094 | NA |
| Notoungulata | Toxodontidae | Toxodontinae | -0.109 | -0.664 |
| Paucituberculata | Caenolestidae |  | -0.853 | -1.066 |
| Peramelemorphia | Chaeropodidae |  | 0.428 | NA |
|  | Peramelidae | Echymiperinae | -0.580 | NA |
|  |  | Peramelinae | 0.019 | NA |
|  |  | Peroryctinae | -0.720 | NA |
|  | Thylacomyidae |  | 0.283 | NA |
| Perissodactyla | Equidae |  | 0.333 | 0.262 |
|  | Rhinocerotidae | Elasmotheriinae | 0.259 | 0.141 |
|  |  | Rhinocerotinae | 0.095 | -0.091 |
|  | Tapiridae |  | 0.188 | -0.008 |
| Pholidota | Manidae |  | 0.650 | 0.519 |
| Pilosa | Bradypodidae |  | 0.922 | 0.720 |
|  | Cyclopedidae |  | 1.561 | 1.518 |
|  | Megalonychidae | Megalonychinae | 0.242 | 0.015 |
|  | Megatheriidae | Megatheriinae | 0.140 | -0.014 |
|  | Mylodontidae | Lestodontinae | -0.102 | -0.262 |
|  |  | Mylodontinae | -0.319 | -0.339 |
|  |  | Scelidotheriinae | -0.003 | -0.121 |
|  | Myrmecophagidae |  | 0.587 | 0.090 |
|  | Nothrotheridae |  | 0.413 | 0.363 |
| Primates | Aotidea |  | 0.179 | 0.081 |
|  | Archaeolemuridae |  | -1.243 | NA |
|  | Atelidae | Alouattinae | 0.386 | 0.273 |
|  |  | Atelinae | 0.021 | -0.260 |
|  | Callitrichidae |  | -0.156 | -0.502 |
|  | Cebidae | Cebinae | 0.532 | 0.441 |
|  |  | Saimiriinae | 0.594 | 0.436 |
|  | Cercopithecidae | Cercopithecinae | -0.069 | -0.374 |
|  |  | Colobinae | -0.413 | -0.759 |
|  | Cheirogaleidae |  | -1.409 | NA |
|  | Daubentoniidae |  | -0.985 | NA |
|  | Galagidae |  | 0.438 | 0.265 |
|  | Hominidae |  | 0.733 | 0.578 |
|  | Hylobatidae |  | -0.442 | -0.766 |
|  | Indriidae |  | -1.601 | NA |
|  | Lemuridae |  | -1.608 | NA |
|  | Lepilemuridae |  | -1.406 | NA |
|  | Lorisidae |  | 0.297 | 0.107 |
|  | Megaladapidae |  | -1.194 | NA |
|  | Palaeopropithecidae |  | -1.384 | NA |
|  | Pitheciidae | Callicebinae | -0.355 | -0.690 |
|  |  | Pitheciinae | -0.299 | -0.573 |
|  | Tarsiidae |  | -0.213 | -0.434 |
| Proboscidea | Elephantidae | Elephantinae | 0.485 | 0.381 |
|  | Gomphotheriidae | Cuvieroniinae | -0.019 | -0.104 |
|  | Mammutidae |  | -0.430 | -0.486 |
|  | Stegodontidae |  | -0.445 | -0.742 |
| Rodentia | Abrocomidae |  | -0.861 | -1.031 |
|  | Anomaluridae | Anomalurinae | 0.578 | 0.083 |
|  |  | Zenkerellinae | 0.061 | -0.454 |
|  | Aplodontidae | Aplodontinae | -0.510 | -0.719 |
|  | Bathyergidae | Bathyerginae | 0.155 | 0.042 |
|  |  | Heterocephalinae | -0.072 | 0.001 |
|  | Calomyscidae |  | -0.487 | -0.421 |
|  | Capromyidae | Capromyinae | -1.556 | NA |
|  |  | Heptaxodontinae | -1.384 | NA |
|  |  | Hexolobodontinae | -1.451 | NA |
|  |  | Isolobodontinae | -1.321 | NA |
|  |  | Plagiodontinae | -1.649 | NA |
|  | Castoridae | Castorinae | 0.650 | 0.687 |
|  |  | Castoroidinae | 0.219 | 0.239 |
|  | Caviidae | Caviinae | 0.072 | -0.085 |
|  |  | Dolichotinae | -0.422 | -0.502 |
|  |  | Hydrochoerinae | 0.151 | 0.015 |
|  | Chinchillidae |  | -1.289 | -1.484 |
|  | Cricetidae | Arvicolinae | 0.336 | 0.184 |
|  |  | Cricetinae | 0.486 | 0.412 |
|  |  | Neotominae | -0.479 | -0.694 |
|  |  | Sigmodontinae | -0.945 | -1.268 |
|  |  | Tylomyinae | -0.411 | -0.633 |
|  | Ctenodactylidae |  | -0.647 | -0.506 |
|  | Ctenomyidae |  | -0.908 | -1.171 |
|  | Cuniculidae |  | 0.724 | 0.583 |
|  | Dasyproctidae |  | 0.307 | 0.128 |
|  | Diatomyidae |  | -1.640 | -1.917 |
|  | Dinomyidae |  | 0.443 | 0.273 |
|  | Dipodidae |  | 0.500 | 0.461 |
|  | Echimyidae |  | -0.307 | -0.618 |
|  | Erethizontidae | Chaetomyinae | -0.413 | -0.586 |
|  |  | Erethizontinae | 0.402 | 0.177 |
|  | Geomyidae |  | -0.235 | -0.449 |
|  | Gliridae | Glirinae | 1.050 | 1.043 |
|  |  | Graphiurinae | 0.488 | 0.413 |
|  |  | Leithiinae | 0.529 | 0.522 |
|  | Heteromyidae | Dipodomyinae | -0.389 | -0.456 |
|  |  | Heteromyinae | -0.488 | -0.640 |
|  |  | Perognathinae | -0.476 | -0.563 |
|  | Hystricidae |  | 0.375 | 0.187 |
|  | Muridae | Deomyinae | 0.251 | 0.162 |
|  |  | Gerbillinae | -0.199 | -0.185 |
|  |  | Leimacomyinae | -1.927 | -2.183 |
|  |  | Lophiomyinae | -1.276 | -1.368 |
|  |  | Murinae | -0.262 | -0.613 |
|  | Myocastoridae |  | -0.431 | -0.547 |
|  | Nesomyidae | Cricetomyinae | 0.838 | 0.427 |
|  |  | Delanymyinae | -0.609 | -0.764 |
|  |  | Dendromurinae | 0.368 | 0.290 |
|  |  | Mystromyinae | 0.172 | 0.154 |
|  |  | Nesomyinae | -1.383 | NA |
|  |  | Petromyscinae | -0.428 | -0.443 |
|  |  | Saccostomurinae | 0.547 | 0.524 |
|  | Octodontidae |  | -1.247 | -1.429 |
|  | Pedetidae |  | 0.346 | 0.201 |
|  | Petromuridae |  | -0.525 | -0.496 |
|  | Platacanthomyidae |  | 0.065 | -0.087 |
|  | Sciuridae | Callosciurinae | -0.218 | -0.478 |
|  |  | Ratufinae | 0.305 | 0.126 |
|  |  | Sciurillinae | 0.855 | 0.764 |
|  |  | Sciurinae | 0.305 | 0.147 |
|  |  | Xerinae | 0.177 | -0.003 |
|  | Spalacidae | Myospalacinae | 0.310 | 0.255 |
|  |  | Rhizomyinae | 0.109 | -0.085 |
|  |  | Spalacinae | 0.084 | 0.044 |
|  | Thryonomyidae |  | 0.702 | 0.636 |
| Scandentia | Ptilocercidae |  | 0.304 | -0.176 |
|  | Tupaiidae |  | 0.137 | -0.039 |
| Tubulidentata | Orycteropodidae |  | 0.522 | 0.450 |

| **Table S6: Determinants of range size in each subfamily, excluding clades endemic to islands or Australia.** For the empirical dataset the mean, standard error and p-value is given based on means weighted by the AIC-weight across all trees. For randomisation, the median and standard error across 1000 simulations along with the two-tailed probability of achieving equal or more extreme values as the empirical dataset in the simulations. For selective removal, we report the largest fraction of species (sorted by range size) that can be removed, while still recovering a significant effect at the same direction as in the empirical dataset. For jackknifing, the median and standard deviation across the 1000 simulations are given with the significance level estimated by the proportion of the simulated datasets having a value of the opposite sign as the median (i.e. the fraction of jack-knife replicates having a negative value if the empirical value is positive or the fraction of jack-knife replicates having a positive value if the empirical value is negative). | | | | |
| --- | --- | --- | --- | --- |
|  | **Empirical** | **Resampling** | **Selective removal** | **Jackknife** |
| **Intercept** | 0.024 (0.091)^NS^ | 0.000 (0.001)^NS^ | NA | 0.044 (0.018)^*^ |
| **Number of species** | 0.844 (0.046)^***^ | 0.810 (0.028)^NS^ | >50% | 0.700 (0.022)^***^ |
| **Body size** | 0.350 (0.064)^***^ | 0.077 (0.052)^***^ | 16% | 0.255 (0.036)^***^ |
| **Annual temperature** | 0.149 (0.070)^*^ | 0.030 (0.072)^NS^ | 24% | 0.148 (0.031)^***^ |
| **Annual precipitation** | -0.017 (0.060)^NS^ | 0.021 (0.081)^NS^ | NA | -0.062 (0.032)^*^ |
| **Temperature width** | 0.278 (0.078)^***^ | 0.166 (0.082)^NS^ | 34% | 0.294 (0.040)^***^ |
| **Precipitation width** | 0.216 (0.060)^***^ | 0.132 (0.080)^NS^ | 5% | 0.168 (0.042)^**^ |
| **Rate of body size evolution** | 0.040 (0.042)^NS^ | 0.013 (0.055)^NS^ | NA | 0.045 (0.021)^*^ |
| **Rate of temperature preference evolution** | -0.062 (0.044)^NS^ | -0.011 (0.064)^NS^ | NA | -0.061 (0.030)^*^ |
| **Rate of precipitation preference evolution** | -0.126 (0.048)^**^ | -0.002 (0.066)^NS^ | 0% | -0.016 (0.030)^NS^ |
| ^NS^ P>0.05 ^*^ 0.01<P<0.05 ^**^ 0.001<P<0.01 ^***^ P<0.001 | | | | |

| **Table S7: Determinants of range size in each subfamily, including all clades.**  For the empirical dataset the mean, standard error and p-value is given based on means weighted by the AIC-weight across all trees. For randomisation, the median and standard error across 1000 simulations along with the two-tailed probability of achieving equal or more extreme values as the empirical dataset in the simulations. For selective removal, we report the largest fraction of species (sorted by range size) that can be removed, while still recovering a significant effect at the same direction as in the empirical dataset. For jackknifing, the median and standard deviation across the 1000 simulations are given with the significance level estimated by the proportion of the simulated datasets having a value of the opposite sign as the median (i.e. the fraction of jack-knife replicates having a negative value if the empirical value is positive or the fraction of jack-knife replicates having a positive value if the empirical value is negative). | | | | |
| --- | --- | --- | --- | --- |
|  | **Empirical** | **Resampling** | **Selective removal** | **Jackknife** |
| **Intercept** | 0.037 (0.145)^NS^ | 0.000 (0.003)^***^ | NA | 0.025 (0.036)^NS^ |
| **Number of species** | 0.717 (0.040)^***^ | 0.810 (0.023)^***^ | >50% | 0.686 (0.026)^***^ |
| **Body size** | 0.283 (0.059)^***^ | 0.068 (0.047)^***^ | >50% | 0.247 (0.039)^***^ |
| **Annual temperature** | 0.149 (0.059)^**^ | 0.037 (0.063)^NS^ | >50% | 0.137 (0.034)^***^ |
| **Annual precipitation** | -0.076 (0.050)^NS^ | 0.004 (0.066)^NS^ | NA | -0.047 (0.034)^NS^ |
| **Temperature width** | 0.274 (0.066)^***^ | 0.169 (0.073)^NS^ | >50% | 0.286 (0.045)^***^ |
| **Precipitation width** | 0.180 (0.051)^***^ | 0.135 (0.073)^NS^ | >50% | 0.174 (0.046)^***^ |
| **Rate of body size evolution** | 0.023 (0.037)^NS^ | 0.021 (0.048)^NS^ | NA | 0.036 (0.023)^NS^ |
| **Rate of temperature preference evolution** | -0.068 (0.038)^*^ | -0.002 (0.054)^NS^ | >50% | -0.064 (0.032)^NS^ |
| **Rate of precipitation preference evolution** | -0.029 (0.044)^NS^ | 0.012 (0.056)^NS^ | NA | -0.010 (0.033)^NS^ |
| ^NS^ P>0.05 ^*^ 0.01<P<0.05 ^**^ 0.001<P<0.01 ^***^ P<0.001 | | | | |

| **Table S8: Determinants of range size in each subfamily excluding bats.**  For the empirical dataset the mean, standard error and p-value is given based on means weighted by the AIC-weight across all trees. For randomisation, the median and standard error across 1000 simulations along with the two-tailed probability of achieving equal or more extreme values as the empirical dataset in the simulations. For selective removal, we report the largest fraction of species (sorted by range size) that can be removed, while still recovering a significant effect at the same direction as in the empirical dataset. For jackknifing, the median and standard deviation across the 1000 simulations are given with the significance level estimated by the proportion of the simulated datasets having a value of the opposite sign as the median (i.e. the fraction of jack-knife replicates having a negative value if the empirical value is positive or the fraction of jack-knife replicates having a positive value if the empirical value is negative). | | | | |
| --- | --- | --- | --- | --- |
|  | **Empirical** | **Resampling** | **Selective removal** | **Jackknife** |
| **Intercept** | 0.072 (0.141)^NS^ | 0.000 (0.004)^***^ | NA | 0.018 (0.037)^NS^ |
| **Number of species** | 0.691 (0.042)^***^ | 0.808 (0.025)^***^ | >50% | 0.684 (0.025)^***^ |
| **Body size** | 0.284 (0.061)^***^ | 0.133 (0.048)^**^ | 5% | 0.240 (0.038)^***^ |
| **Annual temperature** | 0.121 (0.063)^*^ | 0.019 (0.061) ^NS^ | 26% | 0.147 (0.033)^**^ |
| **Annual precipitation** | -0.055 (0.053)^NS^ | -0.023 (0.067)^***^ | NA | -0.056 (0.033)^NS^ |
| **Temperature width** | 0.259 (0.071)^***^ | 0.174 (0.072)^NS^ | 26% | 0.288 (0.040)^***^ |
| **Precipitation width** | 0.192 (0.054)^***^ | 0.110 (0.073)^NS^ | >50% | 0.179 (0.035)^***^ |
| **Rate of body size evolution** | 0.021 (0.039)^NS^ | 0.032 (0.049)^NS^ | NA | 0.036 (0.024)^NS^ |
| **Rate of temperature preference evolution** | -0.046 (0.041)^NS^ | 0.017 (0.054)^NS^ | NA | -0.070 (0.030)^*^ |
| **Rate of precipitation preference evolution** | -0.029 (0.047)^NS^ | -0.001 (0.054)^NS^ | NA | -0.008 (0.032)^NS^ |
| ^NS^ P>0.05 ^*^ 0.01<P<0.05 ^**^ 0.001<P<0.01 ^***^ P<0.001 | | | | |

**Figure S1 Effect of clade age**

Relationship between residuals and (**a**) stem age and (**b**) crown age of the subfamilies, based on the full dataset of 242 subfamilies. The best fitting linear model is plotted.


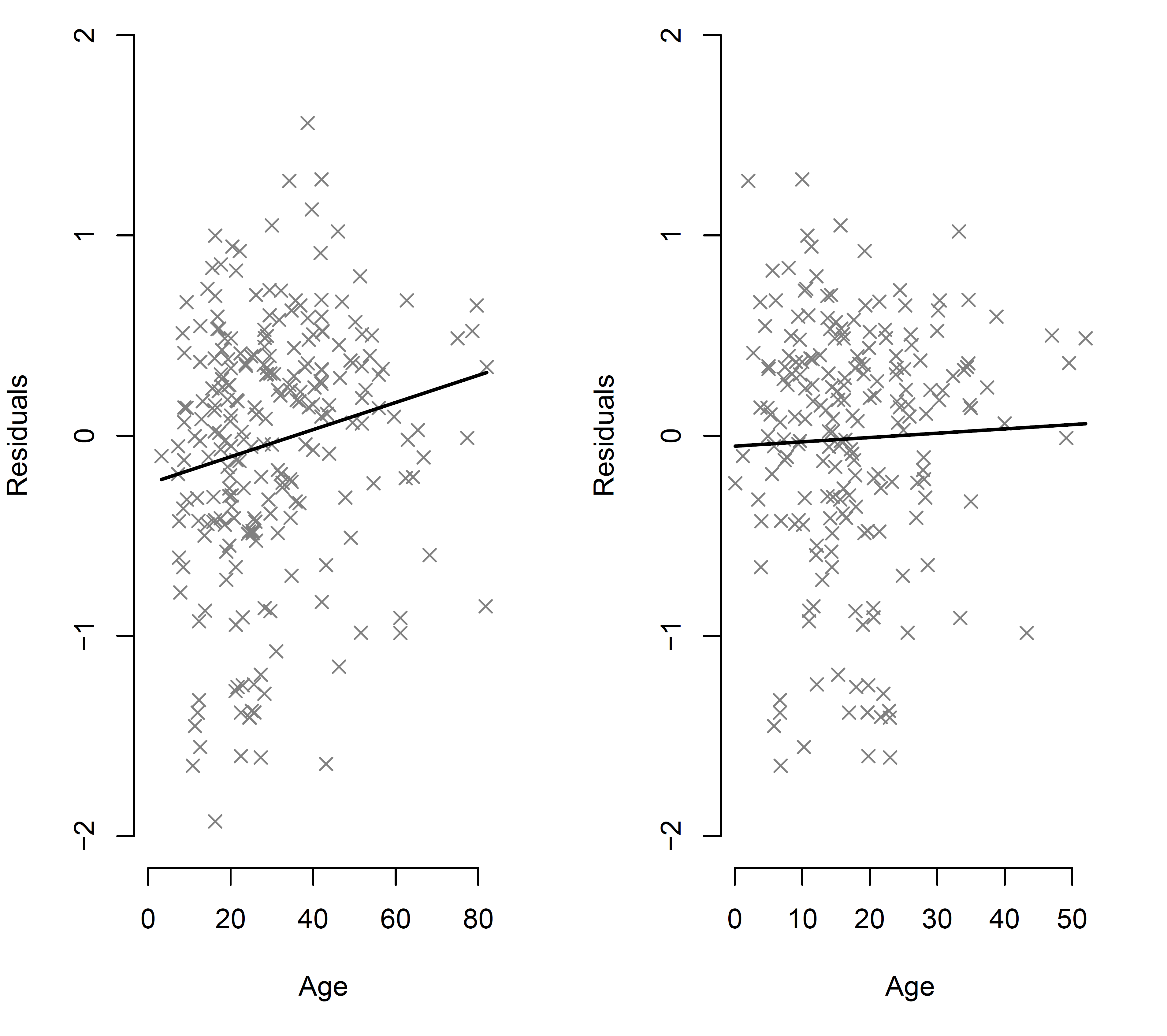


**Figure S2 Relationships for individual predictors**

Relationship between each of the significant predictors and range size. Range size, number of species, temperature niche width and precipitation niche width were log-transformed and both predictors and range sizes were standardised to have a standard error of one and a mean of zero. The best fitting linear model is also plotted, but since this is based on a non-phylogenetic rather than PGLS regression the slope and direction may be different than the results from the full analysis.


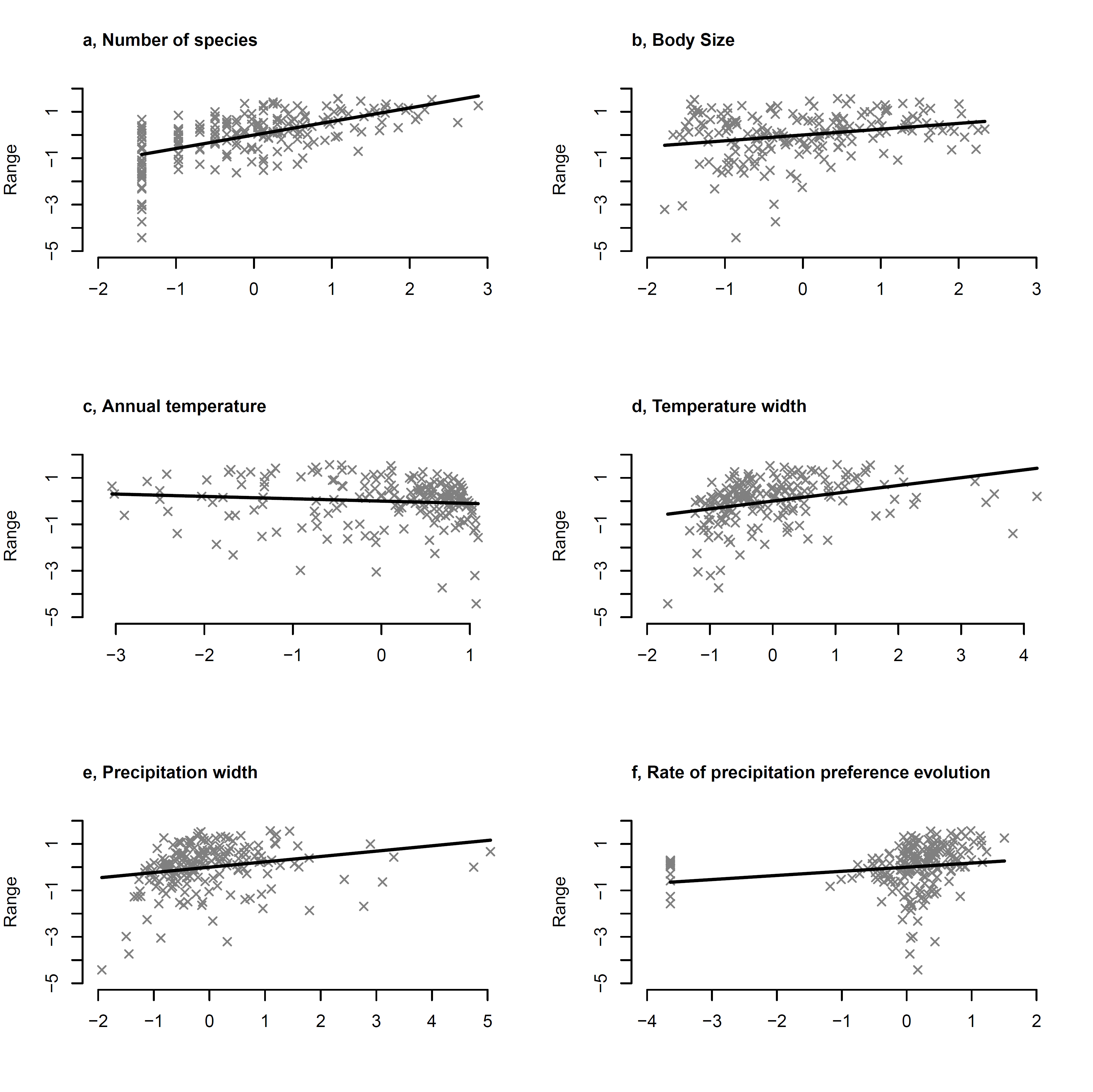


**Figure S3 Evolutionary success**

Results of FiSSE analyses estimating the importance of each of five potential factors (annual precipitation, annual temperature, body size, precipitation niche with and temperature niche width) on net diversification rates of mammals For all analyses species are grouped into two equal sized groups based on having values of the analyzsed factor higher or lower than the median value and the analyses focussed on differences in diversification rates between species from these two groups. **a-b)** shows the pattern for all (non-marine) mammals, **c-d)** the pattern excluding all bats and **e-f**) the pattern excluding island endemic clades. **a**, **c**, and **e)** Difference in diversification rate for species from dry or cold regions, small species or species with narrow niche widths relative to the remaining species across 100 trees. **b**, **d** and **f)** Significance of the difference for each tree. A median value below the stippled line at p=0.05 suggests that the relationship is significant for the majority of trees. The limit of the boxes are the upper and lower quartiles, with the median shown with a thick line. Whiskers extend to the median of the distribution in question plus/minus 1.5 times the inter quartile range; outliers are shown as circles.


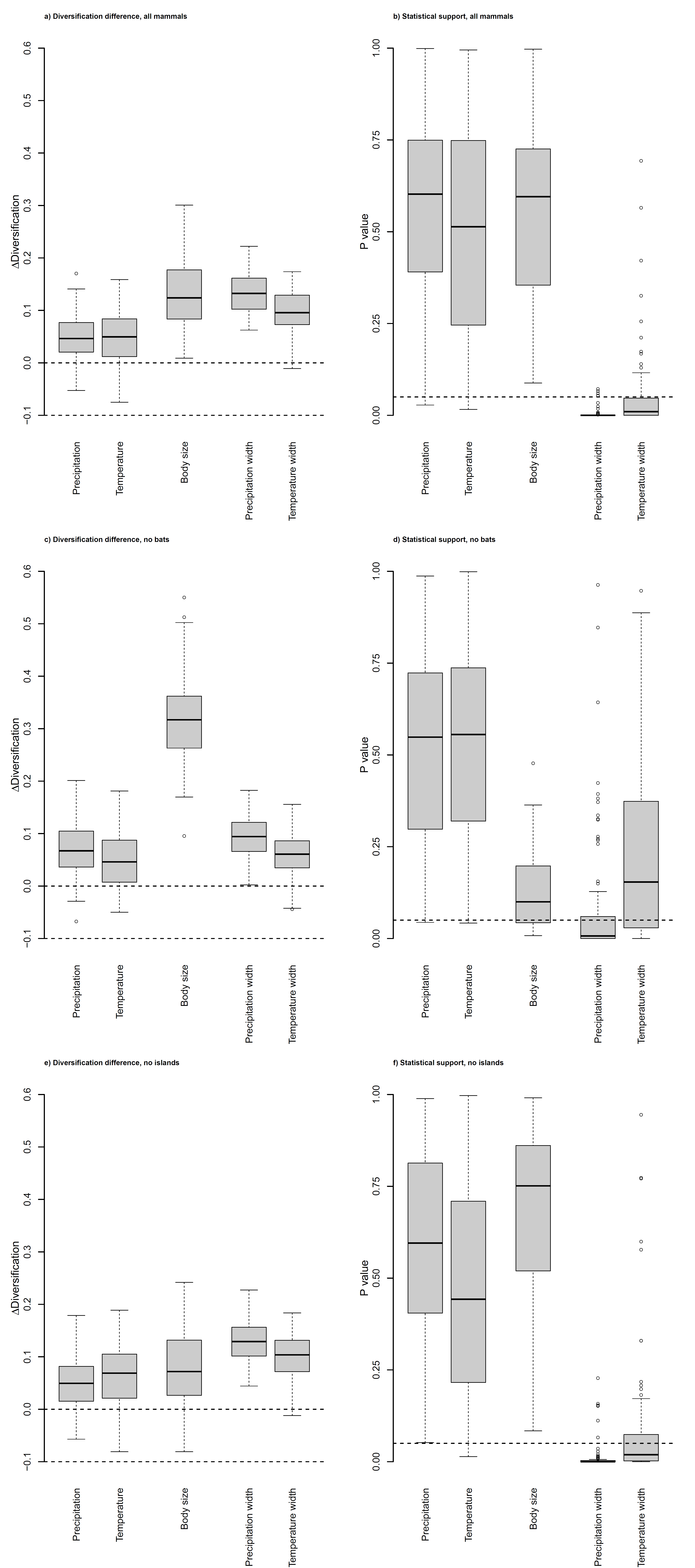

Supplement: Supplementary file 4 [file JBI-45-2227-s004.docx]
